# Role of Phytoconstituents as PPAR Agonists: Implications for Neurodegenerative Disorders

**DOI:** 10.3390/biomedicines9121914

**Published:** 2021-12-14

**Authors:** Sanjay, Anshul Sharma, Hae-Jeung Lee

**Affiliations:** 1 Department of Food Science and Biotechnology, Gachon University, Seongnam-si 13120, Gyeonggi-do, Korea; sanjay.monga4@gmail.com; 2 Department of Food and Nutrition, College of Bionanotechnology, Gachon University, Seongnam-si 13120, Gyeonggi-do, Korea; 3 Institute for Aging and Clinical Nutrition Research, Gachon University, Seongnam-si 13120, Gyeonggi-do, Korea

**Keywords:** neurodegenerative disorders, PPARs, phytoconstituents, bioactivities, anti-inflammation, Alzheimer’s disease, Parkinson disease, Huntington disease

## Abstract

Peroxisome proliferator-activated receptors (PPAR-γ, PPAR-α, and PPAR-β/δ) are ligand-dependent nuclear receptors that play a critical role in the regulation of hundreds of genes through their activation. Their expression and targeted activation play an important role in the treatment of a variety of diseases, including neurodegenerative, cardiovascular, diabetes, and cancer. In recent years, several reviews have been published describing the therapeutic potential of PPAR agonists (natural or synthetic) in the disorders listed above; however, no comprehensive report defining the role of naturally derived phytoconstituents as PPAR agonists targeting neurodegenerative diseases has been published. This review will focus on the role of phytoconstituents as PPAR agonists and the relevant preclinical studies and mechanistic insights into their neuroprotective effects. Exemplary research includes flavonoids, fatty acids, cannabinoids, curcumin, genistein, capsaicin, and piperine, all of which have been shown to be PPAR agonists either directly or indirectly. Additionally, a few studies have demonstrated the use of clinical samples in in vitro investigations. The role of the fruit fly *Drosophila melanogaster* as a potential model for studying neurodegenerative diseases has also been highlighted.

## 1. Introduction

Nuclear receptors (NRs) are a class of ligand-dependent transcription factors that govern a variety of biological processes, such as cell proliferation, growth, reproduction, metabolism, and inflammation when activated by a specific ligand [1,2]. This superfamily of NRs includes peroxisome proliferator-activated receptors (PPARs), steroid hormone receptors, thyroid hormone receptors, vitamin D3 receptors, and retinoic acid receptors [3]. However, ligands of some NRs are yet to be discovered, earning them the moniker “orphan receptors” [4]. Recent research on NRs has revealed that when these receptors bind to specific ligands, they undergo conformational changes, bind to specific DNA sequences throughout the genome, and activate or inhibit target gene expression [4,5,6,7]. Moreover, NRs are known to regulate hundreds of cellular pathways and hence their activity could influence the pathophysiology of a range of diseases [8,9,10]. Their role in disease regulation has piqued the interest of researchers, prompting them to investigate these receptors in relation to various diseases and discover novel natural or synthetic compounds that could either act as agonists or antagonists of these receptors, thereby regulating their altered function in various diseases. Among the various NRs investigated thus far, PPARs have been shown to play an important role in various diseases such as neurodegenerative, inflammatory, and cardiovascular disorders, as well as diabetes and cancers. Several reviews have been published in recent years describing the therapeutic potential of natural and/or synthetic PPAR agonists in a variety of disorders, including neurodegenerative, cardiovascular, diabetes, and cancer. However, a detailed report outlining the role of naturally produced phytoconstituents as PPAR agonists for neurodegenerative disorders has yet to be published. Thus, the aim of this review is to provide a comprehensive discussion of phytoconstituents as PPAR agonists and their importance in neurodegenerative disorders. 

## 2. PPARs Isoforms and Their Distribution across the Nervous System

PPAR gamma (PPARγ), PPAR alpha (PPARα), and PPAR beta/delta (PPAR β/δ, also known as PPARδ) [11], are three isoforms of PPARs that are expressed throughout the brain during development, although PPARα and PPARγ expression levels decline over time and become limited to specific brain locations [12].

PPARα (52 kDa) is primarily found in the basal ganglia, reticulum formation, certain mesencephalic, thalamic, and cranial motor nuclei, and the spinal cord [13]. Additionally, it has also been found in dopaminergic and spiny neurons in the substantia nigra and dorsal striatum, and is vital for controlling behavioral responses [14,15,16]. PPARα has also been discovered in oligodendrocytes, microglia, and astrocytes [17,18,19]. Previous studies have suggested that PPAR activation contributes to neuroprotection by delivering antioxidative and anti-inflammatory actions [20,21]. 

PPARγ (58 kDa) is found in various parts of the brain, including the ventral target area, nucleus accumbens, amygdala, and hippocampus, which are primarily involved in the modulation of reward mechanisms, mood, and learning [13]. PPARγ activation has also been reported to exert neuroprotective and anti-inflammatory effects by inhibiting the nuclear factor kappa-light-chain-enhancer of activated B-cell signaling (NF-κB signaling), activator protein 1 (AP-1), signal transducer and activator of transcription (STATs), and inducible nitric oxide synthase (iNOS) [22]. PPARγ is the most extensively researched PPAR isoform [23].

PPARβ/δ (50 kDa) is widely expressed, and its bioactivity is coordinated with that of PPARα and PPARγ. PPARβ/δ is expressed in neurons, astrocytes, oligodendrocytes, and microglial cells, according to recent studies [22,24]. Similar to the other two isoforms of PPARs, PPARβ/δ have been reported to have anti-inflammatory, antioxidant, and anti-cancer properties. They also play an important role in the regulation of cell cycle and cell death inhibition. However, when compared to the other two PPAR isoforms, PPARβ/δ have received lesser attention [25,26].

## 3. PPARs-Structure and Mechanism of Action

**Figure 1:**
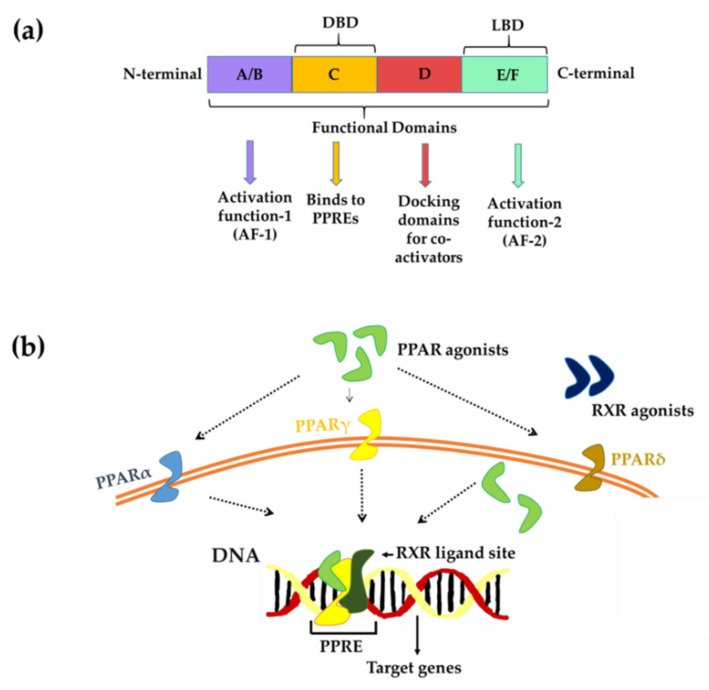
Structure and mechanism of PPARs. (**a**) Three-dimensional structure of PPAR that is composed of four different domains A/B, C, D and E/F; (**b**) upon binding with the specific ligands, these PPARs undergo conformational changes, bind to specific DNA sequences throughout the genome and activate or repress the target gene expression that gives beneficial effects. DBD, DNA-binding domain; LBD, ligand-binding domain; LBP, ligand-binding pocket; AF-1, activation function-1; PPRE, peroxisome proliferator response element; AF-2, activation function-2; PPAR, peroxisome proliferator-activated receptors; PPARα, peroxisome proliferator-activated receptor-α; PPARγ, peroxisome proliferator-activated receptor-γ; PPARδ, peroxisome proliferator-activated receptor-δ; RXR, retinoid X receptors.

PPARs have a three-dimensional structure that comprises four different domains: (1) A/B contains the ligand-independent activation function 1 (AF1) and is involved in transcription activation; (2) C is a DNA-binding domain that binds to peroxisome proliferator response elements (PPRE); (3) D controls the receptor’s ability to bind to DNA and co-repressor binding; and 4) the carboxy-terminal E/F domain is a ligand-binding domain (LBD) containing a ligand-binding pocket (LBP), a ligand-dependent activation function (AF-2), and a region for heteromerization with retinoid-X-receptor (RXR) (Figure 1a) [27,28]. 

PPARs can regulate transcriptional activity directly through two mechanisms. In the first mechanism, PPARs, as a ligand-dependent transcription factor, bind to DNA in the promoter region of genes with sequences known as PPREs. PPAR needs to form a heterodimer with RXR to bind to specific DNA sequences (PPRE) in the promoter region of target genes. This heterodimer exists in a repressor form until the presence of its ligand and can be activated either way, through RXR agonist and PPAR agonist, and the presence of both ligands shows a synergistic effect [29]. Second, transcription factors influence gene expression by interacting with activator proteins, independent of PPREs [30] (Figure 1b). Thus, PPARs can regulate the expression of various genes involved in metabolic pathways. Additionally, PPAR activity can also be controlled by various post-translational modifications such as phosphorylation, small ubiquitin-like modifier (SUMO) phosphorylation, ubiquitination, acetylation, and O-linked-N-acetylglucosaminylation (Figure 1b) [31]. Although they are co-expressed in several different tissues or organs, each possesses its own ligand specificity and functional characteristics. They can sense a variety of endogenous lipids such as unsaturated, mono, and polyunsaturated fatty acids, as well as natural exogenous compounds [32].

## 4. Neurodegenerative Disorders (NDDs) and Role of PPARs

Neurodegenerative disorders are a serious threat to human health. These age-related diseases have become more common owing to an increase in the senior population in recent years [33]. Neurodegenerative disorders, characterized by slow and cumulative neuron loss and neuronal function decrease, frequently result in cognitive and behavioral dysfunction, as well as a significant reduction in the quality of life of the patient. Examples include neuroinflammation, cerebral ischemia, Alzheimer’s disease (AD), multiple sclerosis (MS), Parkinson’s disease (PD), Huntington’s disease (HD), and autoimmune encephalitis (AE) [34]. Drugs for the treatment of neurodegenerative disorders have limited efficacy because of their complicated origins and pathophysiology. Multiple risk factors, including environmental and genetic factors, contribute to vulnerability, in addition to aging.

Neuroinflammation is a term used to describe the complex immune system defense mechanism used in response to several stimuli in the central nervous system. It is marked by the activation of astrocytes and microglia and local immune cell invasion by the release of cytokines and pro-inflammatory molecules, which reverts to normal with the aid of endogenous anti-inflammatory cytokines [35,36]. Under normal circumstances, neurons and astrocytes regulate microglial activation [37]. However, under pathological conditions, including neuronal damage or cell death, repressive action is rendered ineffective. Neuroinflammation mediated by microglia is a key feature of neurodegenerative diseases [38,39], and substantial evidence suggests that inflammation pathways play an important role in the etiology of neurodegenerative disorders [12]. Neuroinflammation also impairs the ability of the brain to generate new neurons [40]. Thus, neuroinflammation could be a potential target for treating neuroinflammatory/neurodegenerative diseases, and several therapeutic approaches may be targeted to mitigate these deleterious effects. 

Several research and review articles published in recent years have provided information on different naturally isolated and identified PPAR agonists that have been investigated and studied for their specific receptors targeting different disorders [41]. The existence, location, and neuroprotective function of PPARs in the CNS were thoroughly addressed by Zolezzi et al. [42]. All three PPARs were found to have distinct effects on the CNS. For example, PPARα, has been related to neuroinflammation and ischemia/reperfusion in certain studies and has been shown to be expressed in diverse regions of the CNS [43,44]. Several in vitro and in vivo studies involving PPAR agonist/antagonist and knockout mice have revealed the significant role of PPARα in anti-inflammation and neuroprotection [45]. PPARγ has been associated with anti-inflammatory activity and brain metabolite balance [46,47,48]. It is believed to induce neuronal differentiation and neutrite outgrowth [49,50]. Because of their diverse roles discovered in various studies, they have been suggested as therapeutic agents in the treatment of CNS disorders such as AD, PD, HD, ischemia/reperfusion injury, and depression [43,51,52,53]. However, only a few studies have focused on the role of PPARβ/δ, which represents their antioxidative and anti-inflammatory functions [54]. As PPAR agonists have gained significant attention in clinical trials and research, the focus of this review will be on phytoconstituents that exhibit direct or indirect PPAR activity [55]. It is crucial to remember that both antagonists and inverse agonists are potentially helpful novel medications with clinical potential. 

### 4.1. Cerebral Ischemia

Ischemic stroke is one of the leading causes of death and disability in developing countries [56]. Ischemic stroke was found to affect approximately 77.2 million people in 2019 [57]. It is a condition caused by an artery blockage that restricts oxygen-rich blood from reaching the brain, resulting in brain tissue injury. Blockages need to be monitored as cerebral ischemia is often caused by blood clots. Neuroinflammation is considered one of the major causes of cerebral ischemia-induced brain damage that is initiated in response to ischemic stroke. Over-activation of microglial cells releases a large number of pro-inflammatory cytokines that aggravate cerebral ischemia-reperfusion injury [58,59]. It has been well documented that PPARγ agonists show an evident protection against cerebral ischemia in rodents by decreasing the apoptotic rates. In addition to this, PPARγ antagonism resulted in increased ischemic infarct [60,61]. It was observed that the mRNA and protein expression levels of PPARγ were increased in cerebral ischemia [62], which obtained a maximum level within 24 h, and could still be perceived until 14 days following ischemic injury [61]. However, it might possible that increased expression of PPARγ is not functionally important, as cerebral ischemia reduces the PPARγ-DNA binding, but treatment with PPARγ agonists such as 15-deoxy-PGJ2 or rosiglitazone recovers their functionality. The roles of PPARs and PPAR agonists in cerebral ischemia have been well described by some recently published literature [63,64]

### 4.2. Alzheimer’s Disease 

AD is a neurodegenerative, progressive, and irreversible brain disorder that is one of the most common causes of dementia worldwide [65]. It accounts for 2/3 of all cases of dementia, with approximately 50–70% of patients with dementia having AD. People with cardiovascular diseases, hypertension, and diabetes also have a higher risk of developing AD as they grow older later. The exact cause of AD remains unknown; however, two main factors have been identified. They are: (1) accumulation of amyloid beta (Aβ) plaques inside the cerebral cortex; and (2) formation of neurofibrillary tangles of intra-nerve filamentous neurons due to tau (τ) protein phosphorylation [65]. PPARγ agonists such as insulin-sensitizing thiazolidione (TZD) drugs; trolitazone (TGZ) and rosiglitazone (RGZ) have been documented to delay the development of AD and promote cell survival through PPARγ [66,67]. Recent reviews have highlighted the role of PPARs and PPAR agonists in AD [25,42,68]. 

### 4.3. Parkinson’s Disease

PD is the second most common neurological condition that predominantly affects the elderly [69]. More than six million individuals worldwide are reportedly affected by PD [70]. It is characterized by the progressive loss of dopaminergic (DA) neurons in the substantia nigra pars compacta (SNc), a reduction in dopamine production in the striatum, and the development of α-synuclein aggregates in the brain, which contribute to the inhibition of thalamic activity, resulting in sluggish movements and stiff limbs in patients [71]. Although the pathology of PD remains unclear, the death of DA neurons in the disease has been related to abnormal α-synuclein accumulation, oxidative stress production, and an increase in neuroinflammation [72]. Various PPAR agonists such as rosiglitazone, fenofibrate and benzafibrate have been shown to protect nerve cells from inflammation, oxidative stress, and programmed cell death [73]. In addition, some of the studies used non-steroidal anti-inflammatory drugs (NSAIDS) (ibuprofen, indomethacin) [63,74], leukotriene receptor antagonists (montelukast) [75], and physical exercise [76] to fight against neurodegenerative conditions. An in-depth review of the role of PPARs in PD was presented by Behl and team [77]. 

### 4.4. Huntington’s Disease

HD is a neurodegenerative disorder that affects neurons and is marked by ataxia, dementia, and loss of normal cognitive and motor functions [78]. The onset of HD typically begins between the ages of 30 and 50 years. In the European population, approximately 12 in 100,000 individuals have HD [79]. Experts estimate that 1 in every 10,000 people has HD. Juvenile HD occurs in approximately 16% of all cases. HD is not prevalent in any particular population, with all races, ethnic groups, and both sexes being affected (http://www.neurocntr.com/huntingtons-disease.php accessed on 25 September 2021). HD is named after mutations in huntingtin protein, which causes neuronal abnormalities such as axon transportation, dysregulation of calcium homeostasis and signaling, mitochondrial dysfunction, and transcription modification [80].

It is documented that down-regulated PPARγ accounts for dysregulation of energy homeostasis in HD, which makes it more important to consider PPARγ agonists as a po-tential target of HD. It has been shown previously that treatment with TZDs in the HD mouse model results in improvements in motor function, weight loss, and neuro-protective proteins such as brain derived neurotropic factor (BDNF) and Bcl-2, and the formation of HTT aggregates. Not only this, the treatment with TZD improved the availability of PPARγ protein, which further helps in normalizing the expression of the glucose transporter type 4 and PPARγ coactivator-1 alpha genes [81]. Moreover, oxidative stress and mitochondrial dysfunction in striatal cells, which express wild-type (STHdhQ7/Q7) or mutant (STHdhQ111/Q111) huntingtin protein at physiological levels, was prevented by rosiglitazone activation of PPARγ [82]. Additionally, the rosiglitazone treatment restored BDNF deficiency in the cerebral cortex, increased Situin-6 protein levels and prevented PGC-1α reduction in N171-82Q HD mouse brain [83].

### 4.5. Multiple Sclerosis

MS is a chronic inflammatory central nervous system (CNS) disease characterized by demyelination of gray and white matter in the brain and spinal cord, which is the root cause of inflammation in the CNS [84,85]. MS is estimated to affect 2.8 million people worldwide (35.9 per 100,000 population) [86]. MS signs include visual, motor, and sensory disturbances, as well as bowel and bladder autonomic disturbances. Additionally, the inflammatory cells that are affected pass through the blood–brain barrier (BBB), invade the CNS, and cause edema [87]. The role of PPARs in MS has been reported recently [88].

### 4.6. Autoimmune Encephalitis

AE refers to a variety of diseases caused by the immune system of the body through an antigen–antibody reaction, which ultimately destroys the healthy cells and tissues of the CNS. General characteristics of AE include epilepsy, mental illness, and cognitive impairment. It contributes to approximately 8–18.5% of the mortality rate worldwide. Few epidemiological investigations have been conducted for AE; however, data from large-scale epidemiological investigations are lacking, thus far [89]. The PPAR agonist, Moringin has showed the protective effects in EAE through activation of PPARγ and inhibiting the release of inflammatory factors [90,91,92]. Another compound, Urosolic acid, showed anti-inflammatory properties and remyelination in the treatment of MS through the PPARγ/CREB signaling pathway [93]. A comprehensive review of the role of PPAR and their agonists in autoimmune diseases was reported in the year 2020 [94]. 

Other neurodegenerative disorders include depression, brain tumors, and intracerebral hemorrhage. Neuroinflammation, which affects brain cells and tissues, is a common characteristic of all neurodegenerative disorders.

## 5. Literature Search Strategy

A literature search was carried out using Google Scholar, PubMed, and Science direct repositories for related findings between January 2004 and May 2021. The following keywords were used to find the relevant information regarding the role of natural compounds as PPAR agonist in neurological disorder: “Natural compounds as PPAR agonist” or “PPAR Agonist” or “PPAR agonists and Neurodegenerative disorders“ or “Role of natural compounds as PPAR agonist in neurodegenerative disorders” or “PPARs in neurological diseases” or Natural ligands for PPARs” or Effects of natural compounds in neurological disorders” or “Neuroinflammation and PPARs” or “ Anti-inflammatory role of natural compounds in neurological disorders”. There are 282 publications in total in this review paper, comprising 156 research articles and 126 review papers.

## 6. Natural Compounds as PPAR Agonists

PPARα, PPARγ, and PPARβ/δ synthetic agonists have been shown to have protective effects in a variety of neurological illness models. Currently, there are 18 clinically approved synthetic PPAR agonists for illnesses such as diabetes, hyperlipidemia, and heart disease [95]. For NDDs, rosiglitazone, pioglitazone, indomethacin, DSP-8658, thiazolidinedione, ibuprofen, fenofibrate, bezafibrate, GW0742, l-165041, and other synthetic agonists that inhibit disease progression have been reviewed before for PD, AD, HD, and amyotrophic lateral sclerosis (ALS) [96]. To the best of our knowledge, no thorough evaluation of phytoconstituents as natural PPAR agonists and their prospective implications has been published thus far. Therefore, this study is the first to summarize the pharmacological effects of various phytoconstituents on neurodegenerative disease prevention. The neuroprotective properties of these natural agonists might be related to their anti-inflammatory properties and the capacity to regulate cell signaling pathways.

### 6.1. Flavonoids

Flavonoids are a class of natural substances widely found in fruits, vegetables, grains, bark, roots, stems, flowers, tea, and wine [97]. A recent review has meticulously organized and reviewed all studies conducted in recent years on flavonoids and their benefits in various diseases [98]. Additionally, the role of flavonoids in neurodegenerative disorders with regard to neuroinflammation, oxidative stress, and proteolytic stress has been defined in a recent review by Devi et al. [99]. The present review focuses on the PPAR-mediated effects of flavonoids on neurological disorders (Table 1).

#### 6.1.1. Biochanin A

Biochanin A (5, 7-dihydroxy-4’-methoxy-isoflavone, BCA) is one of the major isoflavonoids found in red clover, cabbage, and alfalfa. According to recent studies, BCA has been implicated as an antioxidant, antimicrobial, neuroprotective, anti-cancer, anti-inflammatory, antidiabetic, and hepatoprotective agent [100]. The neuroprotective properties of BCA have been attributed in part to its anti-inflammatory and antioxidant activities, as well as its ability to maintain a redox balance [101,102]. Notably, a recent study [103] discovered the effects of BCA on lipopolysaccharide (LPS)-treated BV2 microglial cells through PPAR; BCA was found to significantly suppress the LPS-stimulated inflammatory markers (nitric oxide, prostaglandin E2, tumor necrosis factor-α (TNF-α), interleukin (IL)-1β, and NF-κB) in a concentration-dependent manner (Table 1). Moreover, it was shown to upregulate PPARγ, and all the anti-inflammatory effects were found to be mediated through PPARγ, as PPARγ antagonist (GW9662) abolished the anti-inflammatory activity of BCA.

#### 6.1.2. Icariin

Icariin (ICA) is a natural flavonoid found in Epimedium brevicornum Maxim (a traditional Chinese herb), and has numerous pharmacological functions, including neuroprotective, cardioprotective, anti-inflammatory, anti-depression, anti-tumor, and antioxidant properties [104]. Xiaong et al. [105] published a report that demonstrated Icariin’s PPAR-mediated inhibitory effects on attenuating cerebral ischemia-reperfusion injury. Sprague–Dawley (SD) rats were pretreated with ICA at different doses, followed by cerebral I/R injury induced by middle cerebral artery occlusion (MCAO) and reperfusion. ICA was found to reduce the increased levels of IL-1β and transforming growth factor (TGF)-β1, which were found to exacerbate infarct severity, indicating that inflammation plays an important role in stroke [106,107,108]. NF-κB transcription factor is usually inactive in the cytoplasm, because it binds to the inhibitory unit inhibitor kappa B (IκB) [109]. IκB is rapidly phosphorylated by IκB kinases in response to stimuli, causing NF-κB to translocate to the nucleus and trigger the transcription of target genes such as IL-1β (inflammatory cytokine) [110]. Pretreatment with ICA inhibited the degradation of IκB-α and subsequent phosphorylation of the p65 subunit of NF-κB. Furthermore, after pretreatment with ICA, PPARα and PPARγ, but not PPARβ, were significantly upregulated in the brain tissue, suggesting that ICA has the ability to treat ischemic stroke in rats (Table 1). Previous studies have shown that ICA has neuroprotective effects in rats [111,112,113,114]. As per the authors, this is the first study to show that both PPARα and PPARγ are involved in the neuroprotective effect of ICA [105]. Wang et al. [115] demonstrated that through the upregulation of PPARγ, the effects of ICA can reduce the classical activation of microglia (M1) and Aβ plaque accumulation in the hippocampus and prefrontal cortex of restraint/isolation-stressed amyloid precursor protein (APP)/PS1 mice.

The preventive advantages of ICA when paired with therapeutic hypothermia (TH) have recently been demonstrated in experimental ischemic stroke [116]. TH has been shown to be a neuroprotective agent in the treatment of stroke. The effect of mild hypothermia on infarct volume, neurological impairment, and brain cell death was found to be enhanced by ICA. It also regulated mild hypothermia effects by lowering apoptosis and TNF-α and IL-6 expression. Furthermore, PPARs/nuclear factor erythroid 2-related factor2 (Nrf2)/NF-κB signaling pathways are known to regulate inflammation and apoptosis in ischemic stroke [117]. The combination treatment inhibited NF-κB activation and upregulated the expression of Nrf2 and PPARs in MCAO rats. In addition, the Janus kinase 2/signal transducer and activator of transcription 3 (JAK2/STAT3) pathway is known to be abnormally activated in stroke [118], and this combination treatment appears to decrease this abnormal activation. Furthermore, the presence of an NF-κB inhibitor (JSH-23), which inhibited the activation of the NF-κB and JAK2/STAT3 pathways, reduced the volume of cerebral infarct and neurological deficits in MCAO rats, indicating that the PPARs/Nrf2/NF-κB and JAK2/STAT3/NF-κB pathways are involved in ICA and mild hypothermia in MCAO rats.

Another study [119] found that Icariside II (derivative of icariin, IC-II) protects rats from brain ischemia-reperfusion injury. Compared to MCAO rats, animals pretreated with IC-II had less neurological impairment and infarct volume. In a dose-dependent manner, IC-II reduced IL-1β and TGF-β1 protein expression, IkB degradation, and NF-κB activation induced by MCAO (Table 1). In the ischemic brain, IC-II increased the expression levels of PPARα and PPARγ, and the study concluded that the effects of IC-II were attributable to the upregulation of PPARα and PPARγ, as well as a reduction in NF-κB activation. In a dementia model generated by chronic cerebral hypoperfusion (CCH), Yin et al. [120] discovered that IC-II can relieve cognitive impairments while blocking the amyloidogenic pathway. CCH has been identified as a significant risk factor for both vascular dementia and Aβ aggregation. The study reported the inhibitory effects of IC-II on decreasing the expression levels of APP and β-site APP cleavage enzyme 1 (BACE1), as well as promoting the expression of disintegrin and metalloproteinase domain (ADAM10), and insulin-degrading enzyme (IDE). IC-II was also observed to enhance the expression levels of PPARα, PPARγ, brain-derived neurotrophic factor (BDNF), tyrosine receptor kinase B (TrkB), and levels of serine/threonine-specific protein kinase (Akt) and cAMP response element-binding protein (CREB) phosphorylation. Similarly, icaritin (a hydrolytic product of the natural product icariin) was found to induce cell cycle arrest and apoptosis in glioblastoma multiforme (GBM) by activating PPARγ (Table 1) [121]. Icaritin inhibited the growth of GBM cell lines (U87MG and T98) in a dose- and time-dependent manner, causing cell cycle arrest at the G1/G0 phase by downregulating critical cell cycle regulatory molecules such as cyclin D1, cyclin-dependent kinase (CDK)4, and CDK6 (Table 1). It was also found to trigger apoptosis by boosting caspase-3 cleavage and suppressing Bcl-2 (B-cell lymphoma 2) protein/Bax (Bcl-2 associated X, apoptosis regulator) expression, both of which are markers of apoptosis. Furthermore, icaritin increased the levels of PPAR mRNA expression in GBM cells, and the PPAR antagonist GW9662 reversed the positive effects of icaritin. Adenosine monophosphate-activated protein kinase (AMPK) signaling has also been shown to be involved in PPAR expression. These findings suggest that ICA, as well as its derivatives, can act as PPAR agonists and aid in the treatment of neurodegenerative diseases.

#### 6.1.3. Luteoloside

Luteoloside or cynaroside, commonly known as luteolin-7-glucoside, is another flavonoid found in many plants and herbs. It, together with its metabolites, has been described as an antioxidant, glycolipid balancing, neuroprotective, hepatic metabolism homeostasis, antiobesity, antidiabetic, cardioprotective, and cholesterol-level maintenance agent [122]. The involvement of the PPAR/Nrf2/ NF-κB signaling pathway in attenuating neuroinflammation in localized cerebral ischemia in rats was discovered by Li et al. [117]. As a result of NF-κB signaling inhibition in the brain tissue of MCAO rats, luteoloside decreased neuroinflammation by downregulating IL-1β, TNF-α, iNOS, and cyclooxygenase-2 (COX-2) (Table 1). The anti-inflammatory and neuroprotective actions of PPARγ and Nrf2 have been linked.

#### 6.1.4. Naringenin

Naringenin (NAR), a flavonoid found in citrus fruits and herbs, has been investigated for its antioxidant properties, as well as its ability to enhance glucose metabolism, immune system modulation, and anti-atherogenic, anti-inflammatory, anti-proliferative, antimicrobial, and anti-carcinogenic properties [123]. The neuroprotective roles of naringenin have been described in an animal model of AD, where pretreatment bio-compounds attenuate Aβ-induced impairment of learning and memory through mitigation of lipid peroxidation and apoptosis, and its beneficial effect is somewhat mediated via the estrogenic pathway [124]. Concerning NAR as a PPAR agonist, two in vivo studies [125,126] have been conducted to investigate the effects of NAR on neurodegenerative diseases and their relationship to PPARγ functioning. 

SD rats were administered intracerebroventricular streptozotocin (ICV-STZ) to induce AD, and the designated doses of NAR were administered orally (Table 1) [125]. Rats treated with NAR were found to have better learning and cognitive performance than the control group. Reduced cerebral glucose use was observed in early AD, which is an indication of declining brain function [127], and a previous study has shown that brain insulin deficiency can result in the increased expression of APP and Aβ accumulation [128]. Moreover, evidence gathered from recent studies reflects the toxic effects of Aβ on promoting insulin resistance [129]. In the study [125], NAR was found to be involved in increasing the levels of insulin (INS) and insulin receptor (INSR) in the cerebral cortex and hippocampus. Furthermore, NAR was found to be reversibly associated with ICV-STZ-mediated τ-hyper-phosphorylation in both the hippocampus and cerebral cortex by suppressing glycogen synthase kinase-3β (GSK-3β) expression [125]. GSK-3β is known to suppress insulin signaling and is involved in the phosphorylation of τ protein in an AD brain [130]. Moreover, the IDE was found to degrade soluble Aβ and regulate extracellular levels of Aβ. IDE activity and protein levels have been reported to decline by approximately 50% in AD [131]. A previous study has also supported the role of PPARγ agonists in Aβ clearance by enhancing the expression of IDE [132]. NAR, as a PPARγ agonist, followed the same trend of increasing IDE and Aβ degradation. 

In another study [126] concerning HD, male SD rats were administered an intrastriatal injection of quinolinic acid (QA) to cause neurotoxicity. The defined doses of naringenin or pioglitazone were administered orally for 28 days. NAR significantly reduced QA-induced neurotoxicity by enhancing locomotor function, rearing, grooming, neurological score, footprint analysis, grip strength, and number of slips. NAR was found to attenuate QA-induced alterations in striatal oxidative stress (superoxide dismutase, glutathione, malondialdehyde, and nitric oxide). The expression of neuroinflammatory markers (TNF-α, IL’s and NF-κB mRNA) was reduced by NAR, making it an anti-inflammatory agent (Table 1). It has also been well documented that mitochondria play an important role in neuronal apoptosis, which is a primitive feature of many neurodegenerative diseases, including HD [133,134]. NAR was found to play a role in apoptosis by reducing changes in Bcl-2, Bax, and caspase-3, which are usually involved in the alteration of plasma membrane phospholipids and condensation of nuclear DNA and DNA fragmentation [135]. In addition, NAR was found to be a significant regulator of striatal PPARγ mRNA expression in the brain, which was thought to be due to improvement in mitochondrial enzyme complexes and anti-apoptotic proteins (e.g., Bcl-2), and restoration of mitochondrial function.

#### 6.1.5. Chrysin

Chrysin is a flavonoid found in considerable concentrations in honey, propolis, and a variety of plant extracts. It has been studied as an antiviral, anxiolytic, antihypertensive, antidiabetic, antioxidant, and anti-cancer agents [136]. Because chrysin is an antagonist of NF-κB and inhibits inducible nitric oxide synthase (iNOS) and cyclooxygenase-2 (COX-2), it has been linked to PPAR [137]. Prior to the study by Xiao et al. [138], there had been no reports on the effect of chrysin on experimental autoimmune neuritis (EAN). To generate EAN, Lewis rats were injected with P0 peptide 180–199 (neuritogenic antigen) in both hind footpads. Except for the control, oral gavage of chrysin was administered for 16 days after the first clinical signs were observed. In this study [138], authors found that chrysin has anti-inflammatory properties by downregulating EAN-induced iNOS and COX-2 expression. It also increased the expression of the anti-inflammatory cytokine IL-4 while suppressing the expression of inflammatory cytokines IL-1β, IL-2, IL-6, IL-12, IFN-γ, and TNF-α (Table 1). While the authors [138] did not provide any proof that chrysin is a PPARγ agonist, earlier investigations into chrysin have shown that PPAR upregulation can cause similar effects.

#### 6.1.6. Cyanidin 3-O-β-Glucopyranoside (Cy-3-G)

Blackcurrant, red cabbage microgreens, cherry, blueberry, black soybean, chokeberry, mulberry, and black elderberry all contain Cy-3-G, a significant flavonoid anthocyanin. The pharmacological role of Cy-3-G as a neuroprotective agent has been studied in various neurodegenerative diseases, including cerebral ischemia, AD, PD, and neurotoxicity caused by scopolamine, ethanol, kainic acid (KA), acrolein, and glutamate. In addition, it has been studied as an anti-aging agent [139]. The neuroprotective properties of Cy-3-G have recently been demonstrated in vivo and in vitro [140]. Cy-3-G was found to inhibit cytotoxicity, reactive oxygen species (ROS) production, and Aβ(25–35) aggregation (Table 1), the main factors involved in the progression of AD [141] and helped in the acquisition of spatial memory. All these effects were found to be mediated through PPARγ, as Cy-3-G was found to upregulate PPARγ, and the presence of a PPARγ antagonist (GW9662) abolished all effects. The neuroprotective effects of Cy-3-G both in vivo and in vitro are mediated by PPARγ upregulation, according to this report.

#### 6.1.7. Galangin

Galangin (3, 5, 7,-trihydroxyflavone) is a polyphenolic compound found in honey and medicinal herbs such as *Plantago major* L., *Alpinia officinarum Hance*, and *Scutellaria galericulata L* [142,143]. Galangin has been shown to have antioxidant, anti-inflammatory, antimicrobial, anti-tumor, anti-hypertrophic scar, antidiabetic, anti-arthritic, hepatoprotective, and vasorelaxant activities. Galangin was also found to have bioactivities in urinary tract dysfunction, cerebral ischemia, Alzheimer’s disease, and obesity-related problems [144].

Choi et al. [145] demonstrated the effects of galangin on LPS-treated BV2 cells (microglia). The pretreated galangin was found to inhibit LPS-induced mRNA expression levels of inflammatory cytokines (TNF-α, IL-1β, and IL-6) and inflammatory markers (iNOS and COX-2), as well as induce the anti-inflammatory cytokine IL-10 (Table 1). Galangin was also found to inhibit microglial activation and inflammatory responses in LPS-induced mouse brains, as it decreased Iba-1(microglia activator), mRNA expression levels of TNF-α, IL-1β, IL-6, COX-2, and toll-like receptor (TLR4) (pattern recognition receptor for LPS) but not TLR2.

Furthermore, authors of the study identified that galangin inhibited NF-κB activity, as well as phosphorylation of p38 mitogen-activated protein kinase (MAPK), c-Jun N-terminal kinase (JNK), and Akt. In activated microglia, these kinases have been shown to modulate iNOS and cytokine expression [110,146]. Galangin inhibits ROS production in BV2 cells, and all nicotinamide adenine dinucleotide phosphate (NADPH) oxidase subunits responsible for microglial ROS production (p67phox and gp91phox) as well as induced hemeoxygenase-1 (HO-1) (Table 1), which acts as an antioxidant and anti-inflammatory modulator in microglia [147]. It also contains binding sites for Nrf2, a key transcription factor that regulates HO-1and antioxidant enzyme gene expression. Furthermore, it was able to induce the reporter gene activity of ARE-luc. CREB-mediated transcriptional activity, which acts as an upstream modulator of HO-1 expression, also increased. Finally, PPARγ and PPRE-luc activity were found to be upregulated, and the existence of the PPARγ antagonist abolished the anti-inflammatory effects of galangin, which was supported by PPARγ knockdown. Consistently, a recent study [148] demonstrated that PPARγ mediates the anti-inflammatory effect of galangin on neuroinflammation by regulating NF-κB, phosphatidylinositol-3 kinase (PI3K)/Akt (Protein kinase B), and PPARγ signaling in polyinosinic-polycytidylic acid-stimulated microglia (Table 1). Galangin was also found to boost PPAR transcriptional activity and expression, and the presence of a PPAR antagonist (T0070907) abrogated the anti-inflammatory effects of galangin. Similar findings were observed in vivo [148]. 

### 6.2. Fatty Acids

Fatty acids (FAs) are dietary components with important structural, metabolic, and physiological functions in the human body. They are a primary source of energy and serve as precursors for signaling molecule synthesis [149] and supply essential components for all the cell membranes. FAs have been found to be excellent therapeutic agents for cardiovascular diseases, diabetes, depression, neurodegenerative and pediatric disorders, and cancers [150,151,152]. Additionally, several studies have revealed their antioxidative and anti-inflammatory properties [153,154,155]. Although previous research used diverse ways to understand the mechanism of action of FAs, the following section focuses on the role of PPARs in fatty acid-mediated bioactivities (Table 2).

#### 6.2.1. Poly/Monounsaturated Fatty Acids (PUFAs/MUFAs)

Docosahexaenoic acid (DHA) is a long-chain polyunsaturated (n-3) fatty acid (PUFA) that plays an important role in brain development and neuroprotection [156]. It is abundant in the nervous system, and recent research suggests that it can be used as a dietary supplement to treat cardiovascular ailments, psychiatric disorders, and neurodegenerative conditions [156,157,158]. In addition, n-3 PUFAs, including docosahexaenoic acid (DHA; 22:6-3) and eicosapentaenoic acid (EPA; 20:5-3), have been found to provide significant benefits in a variety of neurodegenerative disorders such as AD, PD, and experimental autoimmune encephalomyelitis (EAE) [159,160]. The potential of PUFAs to alter transcriptional factor activity has been linked to all of their positive benefits, with PPAR playing a critical role in this mechanism of action. DHA has been shown to bind PPARγ [161,162], and its anti-inflammatory effects have been linked to PPARγ activation [163,164].

Maria et al. [165] investigated the effect of DHA on LPS-treated primary glial cultures generated from the cerebral cortex of 1 day old rats. DHA was found to suppress nitric oxide (NO) generation and iNOS expression, lower IL-6 and TNF-α expression, impaired p38 MAPK signaling, and induced PPAR translocation (Table 2). Bernardo et al. [166] discovered that DHA promoted oligodendrocyte differentiation by PPARγ signaling and preventing TNF-α-dependent maturational arrest. Newborn Wistar rats were used to purify and prepare oligodendrocyte progenitors (OPs). Differentiation was performed using antigenic and morphological alterations. Researchers observed that DHA could accelerate the differentiation process after studying OPs at various stages in serum-free media. In different stages of OP differentiation, the results were assessed both morphologically and through the expression of stage-specific markers (G protein-coupled receptor 17 and myelin basic protein). OP differentiation was found to be mediated by PPARγ since it triggered PPARγ nuclear translocation and was inhibited by pretreatment with the PPARγ antagonist GW9662. Extracellular signal-regulated kinase1/2 (ERK1/2) has also been demonstrated to be required for OP differentiation [167]. Additionally, phosphorylation of ERK1/2 by DHA has been identified (Table 2) [166]. Furthermore, TNF-α is known to affect differentiation and cause death in several cell types, including OL, and it is well documented that it impairs OP viability and prevents OP maturation by altering mitochondrial functions [168,169]. TNF-α induced deleterious effects on OP metabolic activity were shown to be masked by DHA, which resembled recent findings on PPAR agonists [168]. 

Mancera et al. [170] found that DHA in triglyceride (TG) form has neuroprotective effects on both in vitro microglial activation and in AE in mice. In the study, the researchers isolated BV2 cells and splenocytes from C57BL/6J mice, as well as CD4+ T cells. Cells were pretreated with TG-DHA and ethyl ester (EE)-DHA before exposure to LPS and IFN-γ (Table 2). Generally, the role of DHA has been demonstrated as an inducer of apoptosis in cancer cell lines [171]. In contrast, in this study, both forms of DHA increased cell viability, with EE-DHA being slightly less than TG-DHA. Furthermore, TG-DHA suppressed NO synthesis and pro-inflammatory cytokines (TNF-α and IL-6) more effectively than EE-DHA (Table 2). However, DHA treatment increased mouse splenocyte cell viability and inhibited anti-splenocyte proliferation but not CD4+ lymphocytes, confirming a previous finding linking the anti-proliferative effects of TG-DHA and modulation of monocytic-dendritic cell antigen presentation activity [172]. In addition, dietary DHA (primarily 250 mg/kg/day) had a positive impact on the clinical course of EAE, while oral TG-DHA had significant effects on the incidence of EAE. Despite the lack of direct evidence for the PPAR-mediated neuroprotective effects of DHA, the findings corroborated a prior study on PPAR agonists and neuroinflammation [173]. 

Eicosapentaenoic acid (EPA) is another significant PUFA found in fish oil, along with DHA. EPA has been demonstrated to be useful in reducing inflammation and lowering cholesterol levels [174,175]. PUFAs have been linked to improving the prognosis of a variety of chronic inflammatory diseases, including rheumatoid arthritis, IgA nephropathy, inflammatory bowel disease, asthma, and psoriasis [176,177]. EPA has been found to have anti-inflammatory neuroprotective effects in the hippocampus in an animal model of AD [178]. EPA and eicosanoid derivatives such as 15-deoxy-Δ-prostaglandin J (15d-PGJ), a cyclooxygenase (COX) product of arachidonic acid (20:4 n-6, AA), are considered to be the natural ligands of PPARγ [179]. However, no research on the role of PPARγ in astrocyte reactions or specifically in cytokine production has been reported till date. Kawashima et al. [180] were the first to demonstrate the inhibitory effects of IL-6 production on IL-1β stimulated C6 glioma cells through PPARγ. After exposure to IL-1β, C6 glioma cells were treated with various doses of EPA. EPA was observed to reduce IL-1β-induced cytotoxicity and IL-6 expression at both intracellular and extracellular levels (Table 2). Several of these effects were found to be mediated through PPARγ, as the PPARγ antagonist GW9662 blocked all the beneficial effects of EPA. These findings indicate that EPA could play an important role in the suppression of cytokine production in tumors by mediating ligand-specific actions through PPARγ. Another study [181] showed that EPA can induce PPARs (α, β, and γ) and alleviate EAE. Mice were fed a fish oil-free diet with or without a 5% (*w/w*) EPA ester supplement 2 weeks before EAE induction, and CD4T cells were used. The EPA blocked all inflammatory cytokines (IFN-γ and IL-17) in CNS-infiltrating CD4T cells and EPA-fed mice had significantly lower clinical EAE scores than non-EPA mice. Additionally, the expression levels of all PPARs were considerably increased in CNS-infiltrating CD4T cells (Table 2). This study indicated that EPA mediates its beneficial effects through PPARs.

Oxidative stress can damage macromolecules such as DNA, proteins, and lipids, resulting in neurodegeneration in the CNS [181]. The PPAR family is a popular target for ROS control. Satyanarayanan et al. [182] demonstrated the antioxidative effects of EPA, DHA, and melatonin receptor agonist (Ramelteon, RMT) on neuronal SH-SY5Y cells. The neuroprotective effects of various substances were investigated for their ability to rescue or protect cells from H_2_O_2_-induced cytotoxicity, both alone and in combination. RMT alone had preventive effects; however, FLX (fluoxetine) and DHA had rescue effects, and EPA alone had both rescue and preventive effects. The combination of RMT and EPA treatment increased cell viability in a dose-dependent manner. RMT, FLX, EPA, and DHA were examined to determine their role in neuroinflammation. The combinatorial treatment of RMT and EPA augmented the activation and translocation of NF-κB to the nucleus, decreased ROS, and increased the expression levels of tyrosine hydroxylase, c-Fos expression (which are markers of neuronal activity), and PPARγ (Table 2). The combination of RMT and EPA reversed the oxidative and inflammatory pathophysiology of PPARγ.

Similarly, another study demonstrated the antioxidant potential of omega-3 fatty acids in activated BV2 microglial cells and SD rats [183]. Treatment with supplement rich in PUFAs (SRP) increased cell viability and reduced oxidative stress (ROS and NO production) in a dose-dependent manner, suggesting the protective role of SRP in neurodegeneration (Table 2). In addition, enhanced PPARγ expression in LPS-treated BV2 cells and increased expression of PPARγ/1 but not PPARγ/2 in the cerebral cortex of SRP-treated animal models imply that PPARγ is involved in mediating the anti-inflammatory effects of SRP.

Linoleic acid, an omega-6 (ω-6) fatty acid, has been suggested to play a key role in the breakdown of APP via PPAR. As per the report, conjugated linoleic acid lowered BACE1 expression and enhanced soluble APPα expression in SH-SY5Y cells, and these effects were found to be mediated through PPARγ (Table 2) [184]. Among others, arachidonic acid (ω-6 PUFA, AA) has been found in mammalian cells and is a precursor of eicosanoids in arthropods and some eukaryotic microbes. Wang et al. [185] discovered that AA could protect hippocampal slices from glutamate or H_2_O_2_ induced stress, as well as improve Cu/Zn-SOD, which were found to be mediated through PPARγ (Table 2), since a PPARγ antagonist (BADGE) reversed these effects. The effect of stearic acid on oxygen/glucose deprivation (OGD) or glutamate on rat cortical or hippocampal slices was studied by the same research team. These neuroprotective effects are thought to be mediated by PPARγ [186]. Similarly, researchers used stearic acid to assess the antioxidant properties of PPARγ [187]. These findings suggest that PPARγ is an important therapeutic target for neurodegenerative and oxidative stress-related disorders. 

Oleic acid (C18:1, cis-9), another example of MUFA, has been reported to have neuroprotective effects on cerebral ischemia through the PPARγ receptor [188]. The study found that oleic acid treatment resulted in reduced infarct volume and functional deficits in MCAO rats, as well as photothrombosis-induced infarct volume, and 4-VO induced neuronal death (Table 2). In the presence of a PPARγ antagonist (GW9662), the effects were reversed. This clearly indicates the role of PPARγ in oleic acid-induced neuroprotective effects [188].

#### 6.2.2. n-3 Fatty Acid-Rich Linseed Oil

Linseed oil (LSO) is a rich source of α-linolenic acid (ALA), and has been studied for its potential to prevent and treat many diseases, such as cardiovascular diseases, arthritis, diabetes, neurodegenerative diseases, and other inflammatory diseases [189,190].

A formulation enriched with n-3 fatty acids has also been investigated as an antioxidant agent in the treatment of oxidative stress [191]. Rao et al. [192] reported that the protective effects of LSO against pro-inflammatory agents increased by partially hydrogenated vegetable fats (PHVF) in a rat model (Table 2). The authors prepared the AIN-93 purified diet according to the previously defined protocol [193]. It has been reported that dietary trans fatty acids (TFA) present in PVHF render the cell membrane [194], and the cell membrane plays a significant role in macrophage functionality [195]. TFA intake has been shown to enhance low-density lipoprotein (LDL) cholesterol, thus lowering high-density lipoprotein (HDL) and increasing plasma triglyceride levels. LDL is known to oxidize and activate NF-κB, resulting in the formation of ROS [196,197,198], which could be related to PPARγ downregulation [199]. In this study, [192], dietary LSO increased cell membrane fluidity of macrophages in Wistar rats and reduced PHVF-induced pro-inflammatory eicosanoids such as prostaglandin E2 (PGE2), thromboxane B2, leukotriene B4 (LTB4), and leukotriene C4 (LTC4), but increased 6-keto prostaglandin F1α (PGF1α) (as compared to PHVF) and reduced pro-inflammatory cytokines (Table 2). The PPARγ expression level was also increased by combining PHVF and LSO. The study indicated that ALA-rich LSO activated PPARγ, which inhibited the inflammatory eicosanoid pathway, NF-κB p65 transcription, and inflammatory mediator secretion, as well as supporting LSO blending with PVHF.

### 6.3. Cannabinoids (CBs)

*Cannabis sativa* is a flowering herbaceous annual plant that is native to Eastern Asia. Endocannabinoids (endogenous cannabinoids) and synthetic cannabinoid-related compounds are examples of CBs, which are bioactive components of *Cannabis sativa*. CBs have been shown to have therapeutic potential in neurodegenerative disorders such as PD, AD, and MS. In addition, cannabinoids have been described as anti-inflammatory, analgesic, and anti-ischemic agents. There are pathways studied for the CB mode of action in association with CB1 and CB2 receptors, the existence of non-CB1/non-CB2 endothelial CB receptors, or through activation of nuclear receptor PPARs due to their lipophilic nature [200]. Table 3 shows examples of cannabinoids that behave as PPAR agonists.

In a human cell culture model of PD (SH-SY5Y), delta-9-tetrahydrocannabinol (Δ^9^-THC) was found to have PPARγ-mediated neuroprotective properties [201]. In SH-SY5Y cells, Δ^9^-THC alleviated 1-methyl-4-phenylpyridinium iodide (MPP+), MPP+-driven cell death, reduced cleaved caspase 3 and ROS levels, and promoted PPARγ expression (Table 3). PPAR antagonist (T0070907) was reported to disrupt Δ^9^-THC neuroprotective, antioxidant, and apoptotic effects. These effects were not inhibited by CB1 receptor blockade [201]. The study suggested that activation of PPARγ leads to antioxidant mediating neuroprotective effects of Δ^9^-THC. Similarly, Zeissler et al. [202] revealed that Δ^9^-THC had therapeutic effects on MPP+-driven SH-SY5Y cells by restoring mitochondrial biogenesis proteins in a PPAR-dependent manner. It was found to prevent cell death and induce PPARγ, peroxisome proliferator-activated receptor gamma coactivator 1 alpha (PGC-1α), mitochondrial transcription factor (TFAM), and mitochondrial DNA (Table 3). The authors also described that in comparison to pioglitazone, Δ^9^-THC provides neuroprotection through PPARγ-dependent renewal of mitochondrial content, which may be useful in treating PD. 

Another study showed the neuroprotective role of tetrahydrocannabinolic acid (THCA) in HD by activating PPARγ [203]. The researchers purified six phytocannabinoids, including Δ^9^-THC and Δ^9^-THCA from the Cannabis variety MONIEK and CBDA (cannabidiol acid) and CBD (cannabidiol) from SARA, CBG (cannabigerol) and CBGA (cannabigerol acid) from AIDA as per the patented methods reported previously [204].

The research used Neuro-2a (N2a), STHdh^Q7/Q7^, and STHdh^Q111/Q111^ (which expresses either a wild-type or a mutated form of huntingtin protein) cells in vitro and 3-nitropropionic acid (NPA, an irreversible inhibitor of respiratory chain complex 2)-induced C57BL/6 (neuro-degenerated) mouse model. In mice treated with 3-NPA, Δ^9^-THCA acts as a neuroprotective agent via a PPARγ-dependent pathway All compounds had different potencies for activating PPARγ, as Δ^9^-THCA > Δ^9^-THC (dose-dependent but significantly reduced after decarboxylation of extract), CBDA > CBD (only at higher doses), and CBGA had the same potential as CBG. PPARγ ligands have been shown to increase mitochondrial biogenesis in neuronal cells [205]. In this study, Δ^9^-THCA was found to significantly induce mitochondrial mass levels when compared to rosiglitazone, but not Δ^9^-THC, and upregulated PGC-1α, a PPARγ interacting protein that plays an important role in mitochondrial biogenesis and could be a potential target in HD. In addition, Δ^9^-THCA was shown to be more effective than rosiglitazone. In STHdh^Q111/Q111^ cells, Δ^9^-THCA was found to enhance neuronal viability after serum deprivation, and this effect was reversed by a PPARγ antagonist (GW9662). The findings were replicated in another collection of infected N2a cells (with huntingtin polyQ repeats) and exhibited a similar outcome. Δ^9^-THCA also downregulated the expression of inflammatory mediators. In mice treated with 3-NPA, Δ^9^-THCA acts as a neuroprotective agent by preventing microgliosis and astrogliosis and by attenuating the upregulation of pro-inflammatory mediators. When mice were administered the PPARγ antagonist T0070903, these effects were reversed. Overall, this research showed that Δ^9^-THCA is a potential therapeutic cannabinoid with beneficial effects mediated through PPARγ (Table 3).

Another compound, cannabigerol (a major phytocannabinoid with a pharmacological profile relatively similar to Δ^9^-THC and CBD), reduced inflammatory mediators and downregulated Huntington-associated genes. In vitro, CBG treatment activated PPAR in cultured striatal cells in a dose-dependent manner (Table 3) [206].

Among other examples, CBD has been shown to reduce Aβ-induced neuroinflammation and promote hippocampal neurogenesis via PPAR in vitro and in vivo [207]. These findings suggest that CBD may have neuroprotective effects by reducing inflammatory markers such as NO, TNF-α, IL-1β, S100 calcium binding protein B (S100B), p50, p65, and glial fibrillary acidic protein (GFAP) (Table 3). PPAR antagonists (GW9662) confirmed that CBDs inhibited reactive gliosis and promoted neurons in the rat hippocampal region. Scuderi et al. [208] demonstrated that CBD reduces Aβ and APP levels and increases APP ubiquitination in SH-SY5Y^APP+^ cells through PPARγ (Table 3). 

Another study revealed the combined effects of CBD and capsazepine (CPZ) in L-3,4-dihydroxyphenylalanine (DOPA)-induced dyskinesia in mice, indicating their protective role in PD. Orofacial abnormal involuntary movements, L-DOPA-induced dyskinesia, and L-DOPA-induced dyskinesia markers such as p-ERK1/ 2, p-AcH3, and inflammatory markers such as COX-2 and NF-κB were all reduced by the combination treatment (Table 3). Moreover, the use of a PPARγ antagonist (GW9662) was found to block the combinatory effects of reducing L-DOPA-induced dyskinesia [209]. Hind et al. [210] described the protective effects of CBD in an in vitro model of BBB OGD. CBD was found to prevent OGD-induced BBB permeability in human brain microvascular endothelial cells (HBMECs) and human astrocyte co-cultures that mimic the BBB. However, the protective effects were reduced in the presence of a PPARγ antagonist (GW9662) and partly reduced by a 5-HT1A receptor antagonist (WAY100135). CBD was also discovered to minimize cell damage by lowering the levels of lactate dehydrogenase (LDH) and vascular cell adhesion-1 (VCAM-1) (Table 3). Moreover, it was also found that HBMEC monocultures had lower VCAM-1 and higher VEGF levels. Another study confirmed the role of CBD in experimental MS. CBD administration was reported to restore the altered PI3K/Akt/ mammalian target of rapamycin (mTOR) pathway following EAE induction in C57BL/6 mice by increasing phosphorylation of PI3K, Akt, and mTOR, activating BDNF and PPARγ, and lowering pro-inflammatory cytokines IFN-γ and IL-17 (Table 3). Suppressing JNK and p38 MAPK has also been found to promote neuronal survival [211]. The above-mentioned studies indicate the role of CBD in PPAR activation, leading to neuroprotective effects.

### 6.4. Other Compounds Extracted from Plants

In addition to flavonoids, FAs, and cannabinoids, other compounds have been extracted from natural species and investigated for their neuroprotective role in neurodegenerative diseases. These compounds are discussed below (Table 4).

#### 6.4.1. Curcumin

Curcumin is a natural polyphenol found in the rhizomatous herbaceous perennial plant *Curcuma* sp. of the ginger family. For several years, *Curcuma* sp. have traditionally been used in Asian countries as medicinal herbs due to their antimutagenic, antimicrobial, anti-inflammatory, and anti-cancer properties. Recent research on *Curcuma longa* and its main constituent curcumin revealed its a wide range of applications in the treatment and prevention of several diseases, which was well studied and reviewed by Sadeghian et al. [212]. Furthermore, it was recently discovered that curcumin has PPARγ agonistic properties in several diseases, including neurodegenerative disorders as described in the following section. 

Chearwae and Bright [213] found that curcumin and 15d-PGJ2 affect TLR4 and TLR9. The authors used EAE, a T-cell-mediated autoimmune model for MS, in their study. EAE was induced in C57BL/6 and SJL/J mice using a standard protocol after they were infected with MOGp35-55 antigens. Both compounds were effective in decreasing the T-cell proliferative response (15d-PGJ2, 46.36%; curcumin, 39.11%) when observed in neural antigen-induced T-cell proliferation. In addition, 15d-PGJ2 and curcumin were able to inhibit TLR4+ and TLR9+ in mice with EAE. Moreover, TLR4 and TLR9 in CD4+ and CD8+ T cells were found to be lower in in vivo and in vitro cultures induced by neural antigens (Table 4). Although no evidence of PPARγ involvement in 15d-PGJ2 and curcumin-mediated effects was found, the results corroborated those of previous studies [214,215].

PPARγ agonists [216] and antagonists [217] have previously been shown to inhibit (by modulating Th1/Th17 responses) and develop (in PPARγ-deficient heterozygous mice) EAE, respectively. Kanakasabai and coworkers [218] provided evidence for the beneficial role of curcumin in EAE by differential regulation of CD4+ T helper cell responses. Curcumin inhibited IFNγ, IL-17 (inhibiting Th1/Th17 responses), and IL-12 family cytokines (IL-12p35, IL-12p40, IL23p19, and IL-27) in the CNS and lymphoid organs of mice, resulting in a decrease in EAE as calculated by clinical score (Table 4). Curcumin was also found to enhance the expression of anti-inflammatory cytokines, including IL-4, IL-40, and PPARγ. Rinwa et al. [219] revealed that curcumin exerts beneficial effects on dementia through PPARγ. Curcumin was found to reverse STZ-induced memory deficits and decrease brain acetylcholinesterase (AChE) activity and oxidative stress (Table 4). All the effects disappeared in the presence of the PPARγ antagonist (BADGE), which was further supported by the decreased performance in the Morris water maze test (MWM) by the STZ+BADGE group as compared to the STZ group. The study presented strong evidence for the mode of action of curcumin as a PPARγ agonist.

Curcumin has been shown to protect neurons during cerebral ischemia in a previous study [228]. Concerning the role of PPAR, a study examined the neuroprotective effects of curcumin in a mouse model of cerebral ischemia-induced inflammation [220]. Curcumin was found to improve neurobehavioral function, reduce infarct volume, minimize neuronal cell damage, suppress neuroinflammatory responses (IL-1β, TNF-α, PGE2, COX-2, and iNOS), and boost PPARγ expression (Table 4). Curcumin was also found to decrease cerebral ischemia-induced IκB degradation and inhibit nuclear translocation of NF-κB and NF-κB-DNA-binding activity. These beneficial effects were found to be abolished in the presence of a PPARγ antagonist, indicating a role of PPARγ in curcumin-mediated neuroprotective effects [220]. Chin et al. [221] demonstrated the role of curcumin in modulating adenosine triphosphate (ATP) concentrations in apoliprotein 4 (APOE4)-targeted replacement mice. While the mechanism through which APOE4 is a risk factor for AD is unknown, it has been discovered to be a key player in multiple pathways that contribute to the disease [229,230]. Mitochondrial dysfunction is one of these pathways and plays a role in key events in the pathogenesis of AD, such as inflammation and cell loss [231]. Curcumin was able to increase adenosine triphosphate (ATP) levels in both of the mouse types as compared to control (control APOE-3 > APOE4) and upregulated the expression of PPARγ-coactivator (PGC1α; control APOE3 > APOE4), GA repeat binding protein alpha subunit (GABP α), PPARγ, and TFAM (Table 4). Protein concentrations of respiratory complexes (1–5) marginally increased in APOE3-targeted mice but not in APOE4-targeted mice. Curcumin increased ATP levels and transcription factors involved in mitochondrial biogenesis, indicating that it has a beneficial function in AD. 

Another study [222] demonstrated that curcumin alleviated the spatial learning and memory deficits in transgenic mice, protected cholinergic neurons in mice and mixed neuronal/glial cultures, and suppressed the neuroinflammatory markers (TNF-α, IL-1β, COX-2, NO, GFAP, and ionized calcium-binding adaptor molecule-1(Iba1)) both in mice and in neuronal/glial mixed cultures. Curcumin also inhibited IkB degradation and NF-κB p65 nuclear translocation, allowing it to suppress NF-κB signaling (Table 4). Curcumin not only increased PPARγ expression, but a PPARγ inhibitor (GW9662) reversed all of the curcumin’s effects, indicating that PPARγ plays a direct role in curcumin-mediated effects both in transgenic mice, and in mixed neuronal glial cultures [222].

Curcumin was also found to promote oligodendrocyte development in a recent study, suggesting that it could be used to treat demyelinating diseases such as MS. Curcumin-treated primary OPs demonstrated increased differentiation and were able to reverse the maturation arrest caused by TNF-α. Curcumin also phosphorylates the protein kinase ERK1/2, which is involved in the regulation of OP transition forms during differentiation. Curcumin was also found to increase the levels of the cofactor PGC1-α and cytochrome c oxidase core protein (COX-1) (Table 4). The benefits of curcumin were mediated through PPAR, as the presence of a PPAR inhibitor (GW9662) abolished curcumin’s effects [223].

#### 6.4.2. Capsaicin

Capsaicin is a naturally occurring compound present in several chili pepper plants. It is present in ample quantities in the placental tissue, but in lesser amounts in the seed and pericarp portions of the plant. Capsaicin has emerged as a therapeutic agent for various diseases in recent years, including cancers, obesity, gastrointestinal disorders, cardiovascular disorders, and dermatological disorders [232].

It has been suggested that capsaicin exerts its effects through specific receptors such as vanilloid receptor-1 (VR-1) [233] and transient receptor potential vanilloid subtype 1 (TRPV1) [234] and, recently, transient receptor potential channel vanilloid subfamily member 1 [235]. However, some studies have described the role of capsaicin in PPARγ. 

In a recent study [224], the anti-cancer properties of capsaicin in glioblastoma cells (LN-18) have been demonstrated. Capsaicin lowered cell survival in a dose-dependent manner and increased PPARγ expression. Thiazolidinediones (troglitazone, rosiglitazone, and pioglitazone) are PPARγ ligands that have been shown to increase cell viability. The effects of thiazolidinediones alone or in combination with capsaicin on LN-18 cells were assessed. Apoptotic indicators such as caspase-9, -8, -3, and cleaved poly (ADP-ribose) polymerase (PARP) were detected in LN-18 cells in the presence of thiazolidinediones alone and in combination with capsaicin, demonstrating that capsaicin potentiates the effects of thiazolidinediones on cell (Table 4).

Although there is clear evidence that PPARγ ligands have anti-proliferative properties in glioblastoma cells, the findings were consistent with previous studies that indicated that PPARγ ligands have anti-proliferative properties in glioblastoma cells [236,237]. Thus, capsaicin-dependent activation of PPARγ leads to enhanced cell death by thiazolidinediones. A recent study [225] found a link between capsaicin consumption and a reduction in brain Aβ and AD-type pathology in vitro as well as in vivo. Capsaicin reduced Aβ40 and Aβ42 levels in a concentration-dependent manner in SH-SY5Y-APP95 cells and prevented cognitive impairment in APP/PS1 mice by inhibiting Aβ deposition and generation in the brain by promoting non-amyloidogenic pathway processing of APP. PPARα levels were also observed to be elevated in both in vivo and in vitro models, showing that PPARα is engaged in ADAM10 (primary α-secretase responsible for APP shedding in the brain)-mediated APP proteolysis. Capsaicin was also shown to reduce τ hyperphosphorylation and neuroinflammation [225].

#### 6.4.3. Piperine

Black pepper contains a large amount of piperine, which is a yellow crystalline alkaloid. Second to curcumin, piperine is a well-known natural compound that has immunomodulatory, anti-carcinogenic, antimicrobial, and anti-ulcer activities [238,239].

The role of the piperamide derivative D4 in neurodegenerative diseases has recently been discovered [226]. Human immortalized astrocytes (SVG) and human immortalized microglial cells (CHME3) were pretreated with D4 and aspirin (Asp) for 2 h followed by post-treatment with LPS. Both D4 and Asp showed cell protective activity in LPS-treated SVG and CHEM3 cells, inhibiting inflammatory cytokines and NF-κB signaling (Table 4), and enhancing PPARγ mRNA expression levels, indicating an indirect involvement of PPARγ in D4-and Asp-mediated neuroprotective and anti-inflammatory effects.

#### 6.4.4. Estradiol

Estradiol, also known as genistein, is a phytoestrogen found abundantly in soya and has been researched as a functional inhibitor of protein kinase and found to play an important role in cardiovascular diseases, obesity, diabetes, cancer, depression, and anxiety [240].

Valles et al. [227] discovered the effects of estradiol/genistein on AD-related neuroinflammation. Primary astrocytes were treated with estradiol/genistein before exposure to β-amyloid. In β-amyloid-treated astrocytes, estradiol or genistein dramatically inhibited pro-inflammatory cytokines and inflammatory markers (Table 4). Furthermore, in the presence and absence of β-amyloid, estradiol/genistein was shown to enhance PPAR expression levels, implying that it plays a role in estradiol/genistein-mediated neuroprotective actions.

## 7. Phytoconstituents’ Other Modes of Action in NDDs

Although this review concentrated on phytoconstituents’ PPAR-mediated bioactivities, there are lot of alternative methods by which these phytoconstituents exert their positive effects in various NDDs. Below is a quick description of the other modes of action of these phytoconstituents, including new findings:

Biochanin A has also been found to provide neuroprotection in glutamate-induced cytotoxicity in PC12 cells by inhibiting apoptosis by decreasing LDH and caspase-3 activity [241], protecting dopaminergic neurons against LPS-induced damage by inhibiting microglia activation and proinflammatory factor generation [242], triggers LPS-induced production of nitric oxide, NF-κB p65, TNF-α, IL-1β, IL-6, PGE2, and ROS in BV-2 cells [243], and alleviating rotenone-induced neurotoxicity in mice by enhancing PI3K/Akt/mTOR signaling and Bcl-1 production [244]. Icariin was found to target Nrf2 signaling to inhibit microglia-mediated neuroinflammation [245], regulating Aβ-enhanced phosphatase and tensin homologue deleted on chromosome 10 protein levels, leading to an improvement in Aβ-induced insulin resistance [246], enhancing neuronal autophagy through the AMPK/mTOR/ autophagy initiating factor uncoordinated-51 like kinase 1 (ULK1) pathway and preventing brain function decline in aging rats [247].

Luteolin-7-O-glucoside was found to protect neurons through activation of the estrogen-receptor-mediated signaling pathway in 1-methyl-4-phenyl-1, 2, 3, 6-tetrahydropyridine-induced mice [248]. Naringenin was shown to target mitochondrial membrane potential, and higher ATP levels and paraquat-induced mRNA expressions of dopamine receptor, dopamine transporter, leucine-rich repeat kinase 2, alpha-synuclein, β-catenin, caspase-3, and BDNF genes [249], and upregulated AMPK-mediated autophagy to prevent neuronal cells from β-Amyloid (1–42) evoked neurotoxicity [250].

Chrysin was found to be involved in alleviating PD pathology as an anti-inflammatory and anti-oxidant agent, together with modifying S100B, BDNF, nerve growth factor (NGF) and glial cell line-derived neurotrophic factor levels [251], Nrf2, which reduces NO, activates myocyte enhancer factor 2D, a critical transcription factor involved in dopaminergic survival. It inhibits MPP-induced upregulation of cleaved-caspase and Bax as well as downregulation of the anti-apoptotic protein Bcl-2 [252], normalizes acetylcholinesterase and butyrylcholinesterase activities, and oxidative damage such as lipid peroxidation, protein carbonylation, catalase, and superoxide dismutase impairment [253]. Cy-3-G was found to regulate Nrf2 mediated oxidative stress [254], and galangin has been shown to suppress protein kinase dependent AP-1, activate forkhead box protein O1, and inhibit LPS-induced MMP-9 expression in rat brain astrocytes [255].

PUFAs were discovered to be involved in cytochrome P450 metabolism in NDDs [256], lysophosphatidylcholine-DHA/EPA-enriched diets were discovered to modulate brain DHA and behavior in mice expressing APOE4 [257], ALA was discovered to suppress GFAP, NF-κB, and IL-1 and modulate Bax, Bcl-2, and caspase-3 activities [258] and induced insulin and insulin-like growth factor I secretion from astrocytes in Aβ-induced SH-SY5Y cells [259]. In addition to these, there are number of studies reported during the recent years, which highlighted the role of natural cannabinoids as well as synthetic cannabinoid receptor agonists in NDDs [260,261].

Other compounds such as curcumin have also been found to modulate lipid peroxidation, HO-1 levels, suppression of malondialdehyde (MDA) levels, increasing glutathione and CAT and Nrf2 activities to produce neuroprotective or anti-ageing effects in different models of NDDs [262], capsaicin decreased brain MDA whereas increased GSH and paraoxonase-1 activity in pentylene-tetrazole-induced Seizures [263], piperamide was found to inhibit NF-κB translocation [264] and activated the Nrf2/HO-1 pathway to induce anti-inflammation in different neuroinflammatory disease models [265]

## 8. In Vitro Studies from Patient’s Sample and Clinical Data 

In addition to in vitro and in vivo research based on phytoconstituents as PPAR agonists, a few studies have evaluated samples from patients with neurodegenerative illnesses to report their effects. Nasl-Khameneh et al. [266] published a study that found that DHA and all-trans retinoic acid (ATRA) had synergistic effects in treating MS. Peripheral blood mononuclear cells (PBMCs) were treated with DHA (15 μM) and ATRA (1 μM) alone and in combination, except for the control (DMSO). Both IL-17 (*p* = 0.02) and RORγt (*p* = 0.01) (transcription factor) were shown to be inhibited by combined treatment with ATRA and DHA, which could be considered as a beneficial outcome, as IL-17 and RORγt were found to be upregulated in MS patients, particularly during clinical exacerbations [267]. Despite the lack of direct evidence for PPARγ participation, the authors postulated that DHA could act as a PPARγ and RXR agonist in their investigation, which was consistent with previous findings [268,269]. These data suggest that PUFAs and vitamin A may be suitable targets for future research and therapeutic intervention efforts. Because this in vitro study was conducted on PBMCs from patients with relapsing-remitting MS (RRMS), more extensive trials will be required to expand these findings.

Fu et al. [270] reported that DHA and Asp have neuroprotective synergistic effects on PD by inhibiting miR-21 and activating RXRα and PPARα. SH-SY5Y cells and PBMCs were collected from blood samples of 15 PD patients. It has been established that miR-21 plays a significant role in human tumor progression and in managing peripheral nerve injury by stimulating axon production [271,272]. The authors’ initial aim was to determine the function of miR-21 in the nervous system. The results showed that the expression of PPARα was substantially lower in PD patients than in the general population, and no significant difference was found with RXRα, and miR-21 was negatively correlated with PPARα. In SH-SY5Y cells, DHA supplementation downregulated miR-21, which upregulated PPARα expression, a transcription factor that stimulates the production of neuroprotective factors such as BDNF and glial derived neurotrophic factor and inhibits NF-κB. According to these findings, decreasing miR-21 levels decreases inflammation and improves neuroprotection. 

In addition to in vitro research using patient samples, the review includes a few clinical investigations of the selected compounds). In a study of 402 patients with mild to moderate AD treated with 2 g of dose/placebo for 18 months, no differences were observed in the cognitive decline in patients with AD [273]. In a 24-wk randomized, double blind, placebo-controlled trial, curcumin C3 complex^®^ 2 gm/day, or 4 gm/day placebo vs. curcumin in patients with mild-to-moderate AD. In this preliminary study, no significant outcomes were reported [274]. Another study evaluated the effect of a commercial product Sativex^®^, a mixture of cannabidiol and Δ^9^-THC on 24 patients with HD for 12 weeks, Sativex^®^ vs. placebo, 12 oral sprays/day. No significant findings were observed [275]. All these studies have been completed. 

## 9. *Drosophila* in Translational Medicine

The genomes of *Drosophila melanogaster* (commonly known as fruit fly) and mammals have a high degree of homology, allowing *Drosophila* to be used in translational medicine. *Drosophila* has been regarded as an excellent model for research in a variety of domains, including medicine, developmental biology, and neurology. The dopaminergic system in *Drosophila* is quite similar to the human system, and dopaminergic cells make up a small percentage of neurons in both the brains of flies and mammals [276]. Two studies have evaluated the importance of *Drosophila* as a model organism for investigating AD [277] and PD [278]. There have also been studies linking the effects of various drugs in a *Drosophila* neurodegenerative model to the activation of PPARs.

In a *Drosophila* model of amyotrophic lateral sclerosis (ALS) based on TDP-43 (TAR DNA-binding protein 43), activation of PPARγ was found to generate neuroprotective benefits [279]. The PPARγ agonist pioglitazone was found to protect *Drosophila* motor neurons and glia against TDP-43-dependent locomotor impairment. It was also found to be neuroprotective in ALS models of *Drosophila* when FUS, rather than SOD1, is expressed in motor neurons. E75 and E78 were identified as in vivo targets of pioglitazone using pharmacogenetic methods. In the context of TDP-43 expression in motor neurons, metabolic methods allowed the identification of several metabolites that pioglitazone can restore. Although PPARγ activation did not prolong longevity in *Drosophila*, it did reveal molecular targets for avoiding locomotor impairment in TDP-43 and FUS models of ALS [279]. 

Overall, the findings from testing PPARs’ natural agonists (such as FAs, flavonoids, curcumin, and others) in various neurological disorders support PPAR-γ, PPAR-α, and PPAR-β (few studies) as potential novel targets for the therapeutic management of common and debilitating conditions such as AD and PD. Few clinical studies have been reported; hence, more clinical trials are needed to prove the efficacy and safety of these natural PPAR agonists. Given the risks associated with synthetic agonist treatment, it is important to investigate alternative administration routes, as well as altering drug doses and delivery. A better understanding of the molecular mechanism of PPAR’s participation in neuroinflammation, which is emerging as a common process in neurodegenerative illnesses, as well as their activation by endogenous ligands, which can also be introduced by food sources, is also required.

## 10. Conclusions and Future Implications

In summary, phytoconstituents as PPAR agonists could be valuable potential therapeutic targets for a variety of neurodegenerative disorders, because data based on in vivo and in vitro models of neurodegenerative disorders support the beneficial effects of PPAR agonists. In addition, it is easy to introduce natural products containing active compounds into the diet because they are common, so they can be used prophylactically throughout life. However, additional research is required to completely understand the potency of PPAR agonists for clinical tests, as well as the processes through which PPAR confers its preventive effects. Nonetheless, as PPAR-based curative treatment cannot entirely slow disease development, blends of other active ingredients with PPAR agonists may provide a superior therapeutic alternative for neuroprotection. Additionally, efforts will need to focus on other phytoconstituents whose pharmacology is currently being investigated. Future research could focus on combining PPAR agonists with a synbiotic and probiotic mixture [280,281], and using the fruit fly (*Drosophila melanogaster*) as a model organism to explore the effects of PPAR agonists [282]. In addition to experimental approaches, multiple methodologies, such as system biology, machine learning, and other bioinformatics tools, can be used to identify and characterize phytoconstituents as new PPAR agonists.

## Figures and Tables

**Table 1:** In vitro and in vivo studies of flavonoids.

Compound Type	Neuro Model	Cell/Animal Type	Treatment	PPAR Type	Outcome	Ref.
Biochanin A	NI	BV2	Pretreatment of Biochanin A (5, 10, 20 μM), 1 h+ LPS (0.5 μg/mL), 24 h	PPARγ	↓iNOS, ↓PGE2, ↓TNF-α, ↓IL-1β, ↓NF-κB	[103]
Icariin	CI/R	SD rats	Pretreated with ICA (10, 30 mg/kg, twice a day), 3 days	PPARα PPARγ	↓IL-1β, ↓TGF-β1, ↓NF-κB, ↑IκB-α, ↑PPARα, ↑PPARγ	[105]
Icariin	AD	APP/PS1 Mice	Control (ddH_2_O) + icariin group (60 mg/kg/day), 6 months	PPARγ	↓IL-1β, ↓IL-6, ↓TNF-α, ↑ IL-4, ↑IL-10, ↑TGF-β1, ↓NF-κB, ↓Aβ42, ↓iNOS, ↓iNOS:Iba1, ↑PPARγ, ↓ memory impair	[115]
Icariin	Experimental ischemic stroke	SD rats	Sham (saline), MCAO + saline, MCAO + MH +saline, MCAO + MH + icariin (60 mg/kg/day), 28 days	PPARα PPARγ	↓TNF-α, ↓IL-6, ↓Bax, ↓cleaved caspase-3, ↑Bcl-2, ↓NF-κB, ↑Nrf2, ↑PPARα, ↑PPARγ	[116]
Icariside II	CI/R	SD rats	Sham (saline), Vehicle (Saline), IC-II (L, H-10, 30 mg/kg), twice a day, 3 days	PPARα PPARγ	↓IL-1β, ↑TGF-β1, ↓IκB-α degradation, ↓NF-κB, ↑PPARα, ↑PPARγ	[119]
Icariside II	VD	SD rats	Sham (saline), BCCAO, BCCAO + ICS II (4, 8, 16 mg/kg/day), 28 days	PPARα PPARγ	Improved learning and memory, ↓neuronal death, ↓Aβ oligomers, ↓APP, ↓BACE1, ↑ADAM10, ↑IDE	[120]
Icaritin	GBM	U87MG, T98G	Icaritin (10, 20 μM), 24 h, 48 h GW9662 (5 μM), 6 h + Icaritin (20 μM), 48 h	PPARγ	↓cell growth, cell cycle arrest at G1/G0 phase, ↓cyclin D1, ↓CDK4, ↓CDK6, ↑apoptosis, ↑caspase-3, ↓Bcl-2, ↑Bax, ↑PPARγ, ↑p-AMPK	[121]
Luteoloside	CI/R	Male Rats	Luteoloside (20, 40, 80 mg/kg) + nimodipine (4 mg/kg)	PPARγ	↑neural function, ↓cerebral edema, ↓IL-1β, ↓TNF-α, ↓iNOS, ↓COX-2, ↓ NF-κB, ↓ p- IκB-α, ↑nuclear Nrf2	[117]
Naringenin	Dementia	SD rats	Control (5 μL saline), STZ (3 mg/kg), NAR-STZ (L-25, M-50, H-100 mg/kg/day), 21 days	PPARγ	Improved learning and memory, ↑INS, ↑INSR, ↓p-Tau, ↓GSK-3β, ↓Aβ levels, ↑IDE	[125]
	HD	SD rats	Control (Saline, 4 μL) + QA (10 mL/kg), NAR + QA (20, 40, 80 mg/kg). Pio (40 mg/kg) + QA, Pio (40 mg/kg) + NAR (80 mg/kg) + QA, 28 days	PPARγ	↓stress, ↓TNF-α, ↓IL-1β, ↓IL-6, ↓ NF-κB ↓Bax-Bcl-2, ↓caspase-3, ↑mitochondrial complex (I-IV) activity, ↑PPARγ	[126]
Chrysin	EAN	Lewis rats	P0 peptide (180–199) 300 μL. Chrysin (50 mg/kg/day), 16 days	PPARγ	↓iNOS, ↓COX-2, ↓NF-κB, ↓IL-1β, ↓IL-2, ↓IL-6, ↓IL-12, ↓IFNγ, ↓TNF-α, ↑IL-4	[138]
Cy-3-G	AD	SH-SY5Y	Control, Aβ (10 μM) 24 h + Cy3G (25 μM), 24 h + GW9662 (20 μM), 3 h	PPARγ	↓cytotoxicity, ↓ROS ↓Aβ (25–35) aggregation, ↑PPARγ	[140]
		APP/PS1 Mice	Cy3G (5 mg/kg/day), GW9662 1 mg/kg/day + Cy3G 5 mg/kg, 2 months		Improved learning and memory	
Galangin	AD	BV2	Pretreated with galangin (10, 30, 50 µM), 1 h followed by LPS treatment (100 ng/mL), 6 or 16h	PPARγ	↓iNOS, ↓IL-1β,↓IL-6,↓TNF-α,↑IL-10,↓NO,↓COX-2 ↓MAPK & NF κB signaling, ↑Nrf2, ↑ CREB, ↑PPAR-γ, ↓NADPH oxidase subunits- p47and gp91, ↑ HO-1	[145]
Galangin	NI	BV2	Galangin pretreatment (0, 10, 30, 50 μM) 1 h + poly(I:C) (10 μg/mL), 6 and 16 h.	PPARγ	↓NO, ↓iNOS, ↓IL-1β, ↓IL-6, ↓TNF-α, ↓ROS, ↓COX-2, ↑IL-10	[148]
		ICR mice	Pretreatment of galangin (50 mg/kg), 4 days + Poly(I:C) (12 mg/kg), 3 h		↓IL-1β, ↓IL-6, ↓TNF-α, ↓iNOS, ↓MMP-8, ↓NF-κB, ↓p-Akt/Akt	

NI, neuroinflammation; LPS, lipopolysaccharides; PPARγ/α, peroxisome proliferator-activated receptor gamma/alpha; iNOS, nitric oxide synthase; PGE2, prostaglandin E2; TNF-α, tumor necrosis factor alpha; IL, interleukin; NF-κB, nuclear factor kappa-light-chain-enhancer of activated B cells; SD, Sprague–Dawley; CI/R, cerebral ischemia/reperfusion studies or cerebral ischemia; ICA, icariin; TGF-β1, transforming growth factor beta 1; IκB-α, inhibitor kappa B-alpha; AD, Alzheimer’s disease; ddH_2_O, double distilled water; Aβ42, amyloid beta-42; Iba-1, allograft inflammatory factor 1; MCAO, middle cerebral artery occlusion; MH, mild hypothermia; Bax, Bcl-2-associated X protein; Bcl-2, B-cell lymphoma 2; Nrf2, nuclear factor erythroid 2–related factor 2, IC-II, icariside II; L, low dose; M, medium dose; H, high dose; VD, vascular dementia; BCCAO, bilateral common carotid artery occlusion, APP, amyloid precursor protein; BACE1, beta-site amyloid precursor protein cleaving enzyme 1; ADAM10, a disintegrin and metalloproteinase domain-containing protein 10; IDE, insulin-degrading enzyme; GBM, glioblastoma multiforme; CDK, cyclin dependent kinase; p-AMPK, phospho-adenosine monophosphate-activated protein kinase; COX-2, cyclooygease-2; NAR, naringenin; STZ, streptozotocin; INS, insulin; INSR, insulin receptor; p-Tau, phosphorylated tau protein; GSK-3β, glycogen synthase kinase 3 beta; HD, Huntington disease; QA, quinolinic acid; Pio, pioglitazone; EAN, experimental autoimmune neuritis; IFN-γ, interferon gamma; Cy3G, cyanidin 3-O-β-glucopyranoside; ROS, reactive oxygen species; Aβ(25-35), amyloid beta (25-35); NO, nitric oxide; MAPK, mitogen-activated protein kinase; CREB, cAMP response element-binding protein; NADPH, nicotinamide adenine dinucleotide phosphate; HO-1, hemeoxygenase-1; poly (I:C), polyinosinic: polycytidylic acid; MMP-8, matrix metalloproteinase 8; pAkt, phosphorylated protein kinase B. ↑, increased expression; ↓, decreased expression.

**Table 2:** In vitro and in vivo studies of fatty acids.

Compound Type	Neuro Model	Cell/Animal Type	Treatment	PPAR Type	Outcome	Ref.
DHA	NI	Primary glial cultures	LPS (10 ng/mL) + DHA (10, 20 μM), 24 h. IFN-γ (200 U/mL) + DHA (10, 20 μM), 24 h	PPARγ	↓NO, ↓iNOS. ↓TNF-α, ↓IL-6, ↑Arg1 activity, ↑IGF-1, ↓p-38 MAPK, ↑ PPARγ, ↑NPC survival ↑NPC differentiation	[165]
	MS	OP	DHA (5, 10 μM), 24 h, PPARγ antagonist GW9662 (1 μM), 30 min	PPARγ	↑OP maturation, ↓ maturational arrest, ↑p-ERK1/2	[166]
	AE	BV2	TG-DHA (1, 5, 10 and 20 μM) or EE-DHA (1, 5, 10, 20 μM), 30 min before LPS (100 ng/mL), 3h and IFN-γ (50 pg/mL), 24 h	PPARγ	↑Cell viability, ↓NO_2_, ↓TNF-α, ↓IL-6 ↓splenocyte proliferation	[170]
		C57BL/6J mice	TG-DHA, (50, 250 mg/kg), or vehicle (0.3% DMSO in water), 56 days		↓ disease severity, improved weight profile	
EPA	Tumor	C6 glioma cells	IL-1β (50 ng/mL), 24 h + EPA (12.5, 25, 50, 100 μM), 24 h	PPARγ	↓IL-1β-induced IL-6	[180]
	EAE	C57BL/6 (B6) mice	Fish-oil-free diet with or without 5% (*w/w*) EPA ester, 2 weeks	PPARαPPARβPPARγ	↓clinical EAE scores, ↓IFN-γ, ↓ IL-17, ↑PPARs	[181]
	Depression	SH-SY5Y cells	H_2_O_2_ (0.07, 0.15, 0.30, 0.62, 1.25, 2.5, 5, 10 μM), agonist (RMT; 10, 20 nM), fluoxetine (5, 10 μM), EPA (30 μM), 24 h	PPARγ	↑cell viability, ↓NF-κB, ↓ROS, ↑cFos, ↑tyrosine hydroxylase ↑PPARγ	[182]
PUFAs	NI	BV2 cells	PUFAs (SRP) 0.5 μg/mL (0.5 μM EPA+ 0.25 μM DHA) to 40 μg/mL (40 μM EPA + 20 μM DHA), 24 h + LPS (1 µg/mL)	PPARγ	↑Cell Viability, ↓ROS, ↓NO, ↑PPARγ	[183]
		SD rats	Control olive oil (2 g/day), SRP (2 g/day), 30 days		↑PPARγ	
CLA	AD	SH-SY5Y cells	CLA (10, 20, 40, 60, 80 μmol/L), 48 h	PPARγ	↓BACE1, ↑sAPPα, ↑PPARγ	[184]
AA	NI/OS	Hippocampal slices from SD rats	Control (artificial CSF immerged), 3 h, glutamate (1 mM), 30min, PUFA (1–10 μM), 30 min + glutamate, H_2_O_2_ (2 mM), 30 min, PUFA (1–10 μM), 30 min + H_2_O_2_, NaN_3_ (10 mM), 30 min, PUFAs (1–10 μM), 30 min + NaN_3_	PPARγ	↓ stress, improved Cu/Zn-SOD activity, ↑CAT	[185]
SA	NI/OS	Brain slices from SD rats	Control (ACSF), OGD (glucose-free ACSF), 2 h, SA (3, 10, 30 µmol/L), 30 min + OGD. Control (ACSF), glutamate (1 mmol/L), 30 min, SA (3, 10, 30 µmol/L), 30 min + glutamate (1 mmol/L), 30 min. Control, NaN_3_ (10 mmol/L), 30 min. SA (3, 10, 30 µmol/L), 30 min + NaN_3_ (10 mmol/L), 30min. BADGE (100 µmol/L), 3 h before	PPARγ	↑ tissue activity	[186]
SA	NI/OS	Cortical neurons from SD rats	SA (3, 10, 30 μM/L), 24 h + glutamate (100 µmol/L), H_2_O_2_ (50 µmol/L), NaN_3_ (20 µmol/L), 24 h. BADGE (100 µmol/L), 1 h	PPARγ	↑Cell viability, ↓lipid peroxidation, ↑SOD, ↑CAT, ↑GSH-Px, ↑PPARγ	[187]
OA	IC/R	SD rats C57BL/6 mice Wistar rats	MCAO model: OA (10, 30, 100 mg/kg), 90 min, edaravone (30 mg/kg), 90 min. Photothrombosis model: OA (20, 60, 200 mg/kg) 24 h after ischemia. 4-VO model: OA (10, 20, 100 mg/kg) 7 days after ischemia. Control, OA (30 mg/kg), GW9661 (4 mg/kg), OA (30 mg/kg) + GW9661 (4 mg/kg), 1 h before MCAO, 24 h.	PPARγ	↓infarct volume, ↓functional deficit, ↓neuronal death, ↓COX-2, ↓iNOS, ↓TNF-α	[188]
LSO	NI	Wistar rats	Supplemented diet with lipids: GNO-10 wt%, PHVF-10 wt%, LSO-10 wt%, PHVF + LSO (2.5, 5.0, 7.5 wt%), 60 days	PPARγ	↓Cholesterol level, ↑cell viability, ↑membrane fluidity, ↓PGE2, ↓6-keto PFA_1__α_, ↓TXB_2_, ↓LTB_4_, ↓LTC_4_, ↓cPLA_2_, ↓COX-2, ↓5-LOX, ↑PPARγ, ↑NF-κB	[192]

DHA, docosahexaenoic acid; NI, neuroinflammation; LPS, lipopolysaccharides; IFN-γ, interferon gamma; PPARγ/α/ β, peroxisome proliferator-activated receptor gamma/alpha/beta; NO, nitric oxide; iNOS, nitric oxide synthase; TNF-α, tumor necrosis factor-alpha; IL, interleukin; Arg1, arginase 1; IGF-1 insulin-like growth factor 1; p38 MAPK, p38 mitogen-activated protein kinase; NPC, neural progenitor cells; MS, multiple sclerosis; OPs, oligodendrocyte progenitors; p-ERK1/2, phosphorylated extracellular signal-regulated kinases 1 and 2; AE, autoimmune encephalomyletis; TG-DHA, triglyceride-DHA; EE-DHA, ethyl ester-DHA; NO2, nitrogen dioxide; DMSO, dimethyl sulfoxide; EAE, experimental autoimmune encephalitis; EPA, eicosapentaenoic acid; H2O2, hydrogen peroxide; RMT, ramelton; NF-κB, nuclear factor kappa-light-chain-enhancer of activated B cells; ROS, reactive oxygen species, cFos, proto-oncogene that is the human homolog of the retroviral oncogene v-fos; PUFA, polyunsaturated fatty acid; SD, Sprague–Dawley; SRP, supplement rich in PUFA; CLA, conjugated linoleic acid, BACE1, beta-site amyloid precursor protein cleaving enzyme 1; sAPPα, soluble amyloid precursor protein alpha; AA, arachidonic acid, OS, oxidative stress; ACSF, artificial cerebrospinal fluid; Cu/Zn-SOD; copper/zinc-superoxide dismutase; CAT, catalase; SA, stearic acid; OGD, oxygen glucose deprivation; BADGE, bisphenol a diglycidyl ether; GSH-Px, glutathione peroxidase; OA, oleic acid; MCAO, middle cerebral artery occlusion; 4-VO, four vessel occlusion; COX-2, cyclooxygenase-2; LSO, linseed oil; GNO, groundnut oil; PHVF, partially hydrogenated vegetable fat; PGE2, prostaglandin E2; 6-keto PFA1α, 6-keto-prostaglandin F1-alpha; TXB, 11-dehydro-thromboxane B2; LTB4/C4, leukotriene B4 or C4; cPLA2, cytosolic phospholipase A2; 5-LOX, 5-lipoxygenase. ↑, increased expression; ↓, decreased expression.

**Table 3:** In vitro and In vivo studies of cannabinoids.

Compound Type	Neuro model	Model	Treatment	PPAR type	Outcome	Ref.
Δ^9^-THC	PD	SH-SY5Y differentiated neuronal cells	MPP+ (5 mM), lactacystin (20 mM), and Paraquat (500 mM), 6, 12, 24, 48, Δ^9^-THC (0.1, 1, 5 ,10 μM), 24 h. PPARγ inhibitor (T0070907; 10 μM), 24 h	PPARγ	↓cell death, ↓cleaved caspase-3, ↓ROS, ↑ PPARγ	[201]
Δ^9^-THC	PD	SH-SY5Y differentiated neuronal cells	MPP+ (7 mM) + Δ^9^-THC (10) µM /pio (5 µM), 48 h. PPARγ inhibitor (T0070907; 10 and 5 µM)	PPARγ	↓cell death, ↑PPARγ, ↑PGC-1α, ↑TFAM, ↑mitochondrial DNA content	[202]
∆^9^-THCA, CBDA, CBG	HD	HEK-293 T Neuro-2a STHdh^Q7/Q7^, STHdh^Q111/Q111^ cells C57BL/6 Mice	N2a cells (Δ^9^-THCA, 0, 0.5, and 1 μM), 48 h. HEK-293 T cells (∆^9^-THCA, CBDA, and CBGA, 0.1–15 μM), 6 h. STHdh^Q7/Q7^ cells (1–10 μM ∆^9^-THCA), 1 h/CB 20 mg/kg, 30 min before 3-NPA, every 24 h, 4 days	PPARγ	↑ neuronal cell viability, ↑ mitochondrial mass, ↓TNF-α, ↓ iNOS, ↓COX-2, ↓IL-6, ↑PPAR-γ expression in HEK-293 T cellsImproved behavioral symptomatology ↓TNF-α, ↓iNOS, ↓COX-2, ↓IL-6	[203]
CBG	HD	STHdh^Q7/Q7^ and STHdh ^Q111/Q111^ cells * C57BL/6 and R6/2 mice	CBG (10 mg/kg), every 24 h, 6 weeks	PPARγ	CBG dose-dependently activated PPARγ ↓neuronal loss, improved motor activities, recovered catalase, SOD, GSH, ↓sgkl, ↓Cd44, ↓COX-2, ↓iNOS, ↓IL-6, ↓TNF-α	[206]
CBD	NI	Primary astrocytes of/and SD rats	Aβ (1 µg/mL), Aβ (1 µg/mL) + CBD (10^−9^–10^−7^ M), Aβ (1 µg/mL) + CBD (10^−9^–10^−7^ M) + GW9662 (9 nM), Aβ (1 µg/mL) + CBD (10^−9^–10^−7^ M) + PPARα (MK886; 3 µM), 24 h. Aβ (30 ng), Aβ (30 ng) + CBD (10 mg/kg), Aβ (30 ng) + CBD (10mg/kg) + MK886 (10 mg/kg, Aβ (30 ng) + CBD (10 mg/kg) + GW9662 (1 mg/kg), 15 days.	PPARα PPARγ	↓NO, ↓iNOS, ↓TNF-α, ↓S100B, ↓IL-1β, ↑GFAP, ↓NF- κB ↓iNOS, ↓NF-κB, ↓S100B, ↑GFAP, ↓calinbindin, ↓gliosis, ↑neuronal survival, ↑neurogenesis	[207]
CBD	AD	SH-SY5Y	SH-SY5Y^empty vector^ and SH-SY5Y^APP+^; CBD (10^-9^–10^-6^ M), CBD (10^-9^–10^-6^ M) + MK886 (3 μM), CBD (10^-9^–10^-6^ M) + GW9662 (9nM), 24 h	PPARα PPARγ	↓APP, ↓Aβ, ↑APP ubiquitination, ↑ PPARγ, ↓apoptosis	[208]
CBD	PD	C57⁄BL6 mice	6-Hydroxydopamine (2.5 mg/mL), L-DOPA (25 mg/kg), 2 injections/day, 21 days with benserazide (10 mg/kg), CBD (15, 30, 60 mg/kg), 3 days 15 min before L-DOPA. CPZ (5 mg/kg) + CBD (30 mg/kg)/L-DOPA and AM251 (1 mg/kg), GW9662 (4 mg/kg), 45 mins	PPARγ	↓orofacial abnormal involuntary movements, ↓ p-ERK1/ 2, ↓p-AcH3, ↓COX-2, ↓NF-κB	[209]
CBD	Ischemia	HBMEC and human astrocyte co-cultures model	CBD (100 nM, 1 μM and 10 μM), 6, 8, 12, 16, 20, 24, 28, 32 h + OGD PPARγ inhibitor (100 nM), similar to CBD	PPARγ	↓BBB permeability, ↓LDH, ↓VCAM-1	[210]
CBD	Experimental MS	C57BL/6 mice	Naïve, EAE, EAE + CBD (10 mg/kg), daily, 14 days	PPARγ	↑histological EAE score, ↑pPI3K/PI3K, ↑pAkt/Akt, ↑pmTOR/mTOR, ↑ pS6K/S6K, ↑BDNF, ↓IFN-γ, ↓IL-17, ↑PPARγ, ↓JNK, ↓ pp38/p38	[211]

* Information not available; Δ9-THC, delta-9-tetrahydrocannabinol; PD, Parkinson’s disease; MPP, 1-methyl-4-phenylpyridinium; PPARγ/α, peroxisome proliferator-activated receptor gamma/alpha; ROS, reactive oxygen species; Pio, pioglitazone; PGC-1α, peroxisome proliferator-activated receptor gamma coactivator 1 alpha; TFAM, the mitochondrial transcription factor; HD, Huntington’s disease; CBDA, cannabidiolic acid; CBG, cannabigerol; CBGA, cannabigerolic acid; 3-NPA, 3-nitropropanoic acid; TNF-α, tumor necrosis factor alpha; iNOS, nitric oxide synthase; COX-2, cyclooxygenase-2; IL-interleukin; CAT, catalase; SOD, superoxide dismutase; GSH, glutathione; sgkl, serine⁄threonine protein kinase, Cd44, multifunctional cell surface adhesion receptor, CBD, cannabidiol; NI, neuroinflammation; Aβ, amyloid beta; NO, nitric oxide; S100B, S100 calcium binding protein B; GFAP, glial fibrillary acidic protein; NF-κB, nuclear factor kappa-light-chain-enhancer of activated B cells; SD, Sprague–Dawley; AD, Alzheimer’s disease; APP, amyloid precursor protein; L-DOPA, L-dihydroxyphenylalanine; CPZ, capsazepine; p-ERK1/2, phosphorylated extracellular signal-regulated kinases 1 and 2; p-AcH3, acetylcholine receptor subunit beta-like 1; BBB, blood brain barrier; LDH, lactate dehydrogenase; VCAM-1, vascular cell adhesion protein 1; MS, multiple sclerosis; EAE, experimental autoimmune encephalitis; pP13K, phospho P13 kinase; pAkt, phosphorylated protein kinase B; pmTOR, phospho mammalian target of rapamycin; pS6K, phospho-p70 S6 kinase; BDNF, brain-derived neurotropic factor; IFN-γ, interferon gamma; JNK, c-Jun N-terminal kinase; pp38/p38 MAPK, phospho p38 mitogen-activated protein kinase. ↑, increased expression; ↓, decreased expression.

**Table 4:** In vitro and in vivo studies of other phytoconstituents.

Compound Type	Neuro Model	Cell/Animal type	Treatment	PPAR Type	Outcome	Ref.
Curcumin/15d-PGJ2	AE	C57BL/6 SJL/J mice	Immunized mice (MOGp35–55 antigen), curcumin or 15d-PGJ2 (100 μg in 25 μL DMSO) alternate day, 14 days	PPARγ	↓TLR4, ↓TLR9 in CD4+ and CD8+ T cells	[213]
Curcumin	AE	C57BL/6 mice	Curcumin (100 µg) alternate day, 14 days. Immunized (MOGp35-55), 36 h. Spleen cells with curcumin (2.5, 5, 10, 25 µM)	PPARγ	↓IFNγ, ↓IL-17, ↓IL-12, ↓IL-23, ↑IL-10, ↑PPARγ ↑ CD4CD25^+^Foxp3^+^ Treg cells	[218]
Curcumin	Experimental dementia	Swiss albino mice	Control (DW; 10 mL/kg, 30 min), artificial CSF control (25 mg/mL, 10 µL), STZ (3 mg/kg, 10 µL) alternate days, 14 days, STZ + curcumin (20 mg/kg), 14 days	PPARγ	Improved learning and memory, ↓AChE activity, ↓oxidative stress, ↑PPARγ	[219]
	CI/R	SD rats	MCAO group, curcumin (200 mg/kg) + MCAO group, curcumin (200 mg/kg) + GW9662 (4 mg/kg) + MCAO group, MG132 + MCAO group, and MG132 alone group. Curcumin + PPARγ inhibitor GW96624 mg/kg, 3 days	PPARγ	↑PPARγ, ↑PPARγ-PPRE binding activity, ↓ infarct volume, ↓neurological deficits, ↓neuronal damage ↓IL-1β, ↓TNF-α, ↓PGE2, ↓NO, ↓COX-2, ↓iNOS, ↓IκB degradation, ↓NF-κB	[220]
	AD	APOE3- and AOE4-targeted gene replacement mice	Two diet groups (control and 0.2 % curcumin-supplemented), 3 months	PPARγ	↑ATP, ↑TFAM, ↑PPARγ, ↑PGC-1α, GABPa, ↑mitochondrial respiratory complex IV in APOE-3	[221]
	AD	Neuronal/glial cells from APP/PS1	Pretreatment curcumin (10 μM), 1 h + Aβ42 (25 μM), 24 h. GW9662 (1 μM) or PPARγ siRNA was transfected 1 h prior to Aβ42 treatment	PPARγ	Improved learning and memory ↑ChAT, ↑Ach ↓LDH, ↓TNF-α, ↓IL-1b, ↓COX-2, ↓NO, ↓GFAP, ↓Iba-1, ↓Mac-1, ↑PPAR-γ, ↑IkB-α expression, ↓ NF-κB p65	[222]
	MS	OPs	OPs were treated with Curcumin (1, 5 μM), 24 h. Curcumin + GW9662 (1 μM pretreatment) 30 min. TNF-α (10 ng/mL), 24 h + curcumin (1 μM)	PPARγ	↑OPs differentiation, ↓TNF-α induced maturation arrest, ↑p-ERK1/ERK2, ↑PGC1-α, ↑COX-1, ↑PPARγ	[223]
Capsaicin	Tumor	LN-18	Capsaicin (25, 50, 100, 200, 300, 400 µM), CPZ (2, 10, 20 µM), 24 and 48 h	PPARγ	↑apoptosis, ↑caspase-9, -8 and -3, ↑PPARγ	[224]
	AD	SH-SY5Y-APP695 cells	Capsaicin (0.1, 1, 5, 10, 50 μM), 24 h	PPARα	↓Aβ40, ↓Aβ42, ↑CTF-α/CTF-β	[225]
		APP/PS1 mice	Capsaicin (30 mg/kg), 6 months		↓cognitive impairment, ↓Aβ40, ↑CTF-α/CTF-β, ↑ADAM10, ↓TNF-α, ↓IFN-γ, ↓IL-6, ↑PSD98, ↑SYN1, ↑Map-2, ↑SNAP25, ↑VAMP1, ↑NeuN, ↑PPARα	
Piperine (D4)	AE	SVG, CHME3	Pretreatment D4 (0.86 μM) and aspirin (50.51μM), 2 h + LPS (100 ng/ml), 24 h	PPARγ	↑cell viability, ↓TNF-α, ↓IL-1β, ↓iNOS, ↓NF-κB, ↑ PPARγ	[226]
Estradiol	AD	Primary astrocytes	Pretreatment 17-βestradiol (0.2 nM) or genistein (0.5 µM), 48 h + 5 μM amyloid beta (Aβ), 24 h	PPARγ	↓TNF-α, ↓IL-1β, ↓COX-2, ↓NO, ↑PPARγ	[227]

AE, autoimmune encephalitis; 15d-PGJ2, 15-Deoxy-∆-12,14-Prostaglandin J2; DMSO, dimethyl sulfoxide; TLR, toll-like receptor; CD, cluster of differentiation; IFN-γ, interferon gamma; IL, interleukin; PPARγ, peroxisome proliferator-activated receptor gamma; Treg, T-regulatory cells; DW, distilled water; CSF, cerebrospinal fluid; STZ, streptozotocoin; AChE, acetylcholinesterase; CI/R, cerebral ischemia/reperfusion studies or cerebral ischemia; MCAO, middle cerebral artery occlusion; PPRE, peroxisome proliferator-activated receptor response element; TNF-α, tumor necrosis factor alpha; PGE2, prostaglandin E2; NO, nitric oxide; COX-2, cyclooxygenase; iNOS, nitric oxide synthase; IkB-α, inhibitor kappa B alpha; NF-κB, nuclear factor kappa-light-chain-enhancer of activated B cells; AD, Alzheimer’s disease; APOE-3/-4, apolipoprotein-3/-4; ATP, adenosine triphosphate; TFAM, the mitochondrial transcription factor A; PGC-1α, peroxisome proliferator-activated receptor gamma coactivator 1 alpha; GABPA, GA binding protein transcription factor subunit alpha; Aβ42, amyloid beta 42; ChAT, choline acetyltransferase; Ach, acetylcholine; LDH, lactate dehydrogenase; GFAP, glial fibrillary acidic protein; Iba-1, allograft inflammatory factor 1; Mac-1, macrophage-1 antigen; MS, multiple sclerosis; OPs, oligodendrocyte progenitors; p-ERK1/2, phosphorylated extracellular signal-regulated kinases 1 and 2; CPZ, capsazepine; Aβ, amyloid beta; CTF- α/β; carboxyterminal fragments generated by α/β-secretase; ADAM10, a disintegrin and metalloproteinase domain-containing protein 10; PSD98, postsynaptic density protein; SYN1, synapsin I; Map-2, microtubule-associated protein 2; SNAP25, synaptosome-associated protein 25; VAMP1, vesicle-associated membrane protein 1 viability. ↑, increased expression; ↓, decreased expression.

## Data Availability

Not applicable.
